# A Synthetic Interaction Screen Identifies Factors Selectively Required for Proliferation and *TERT* Transcription in p53-Deficient Human Cancer Cells

**DOI:** 10.1371/journal.pgen.1003151

**Published:** 2012-12-20

**Authors:** Li Xie, Claude Gazin, Sung Mi Park, Lihua J. Zhu, Marie-anne Debily, Ellen L. W. Kittler, Maria L. Zapp, David Lapointe, Stephane Gobeil, Ching-Man Virbasius, Michael R. Green

**Affiliations:** 1 Howard Hughes Medical Institute, Programs in Gene Function and Expression and Molecular Medicine, University of Massachusetts Medical School, Worcester, Massachusetts, United States of America; 2 CEA/DSV/iRCM/LEFG, Genopole G2, Evry, France; 3 INSERM U967 and Université Paris Diderot, Evry, France; 4 Programs in Gene Function and Expression and Molecular Medicine, University of Massachusetts Medical School, Worcester, Massachusetts, United States of America; 5 Université d'Evry Val d'Essonne, Evry, France; 6 Program in Molecular Medicine and Center for AIDS Research, University of Massachusetts Medical School, Worcester, Massachusetts, United States of America; 7 Department of Cell Biology, University of Massachusetts Medical School, Worcester, Massachusetts, United States of America; Fred Hutchinson Cancer Research Center, United States of America

## Abstract

Numerous genetic and epigenetic alterations render cancer cells selectively dependent on specific genes and regulatory pathways, and represent potential vulnerabilities that can be therapeutically exploited. Here we describe an RNA interference (RNAi)–based synthetic interaction screen to identify genes preferentially required for proliferation of p53-deficient (p53−) human cancer cells. We find that compared to p53-competent (p53+) human cancer cell lines, diverse p53− human cancer cell lines are preferentially sensitive to loss of the transcription factor ETV1 and the DNA damage kinase ATR. In p53− cells, RNAi–mediated knockdown of ETV1 or ATR results in decreased expression of the telomerase catalytic subunit TERT leading to growth arrest, which can be reversed by ectopic *TERT* expression. Chromatin immunoprecipitation analysis reveals that ETV1 binds to a region downstream of the *TERT* transcriptional start-site in p53− but not p53+ cells. We find that the role of ATR is to phosphorylate and thereby stabilize ETV1. Our collective results identify a regulatory pathway involving ETV1, ATR, and TERT that is preferentially important for proliferation of diverse p53− cancer cells.

## Author Summary

The conversion of a normal cell into a cancer cell involves activating genes that promote cancer growth (oncogenes) and/or inactivating genes that normally act to inhibit cancer growth (tumor suppressor genes). The tumor suppressor gene *p53* is the most frequently mutated gene in human cancers, being inactivated in approximately half of all tumors. In addition, loss of p53 function is often associated with increased resistance to chemotherapy and/or poor survival. For these reasons, the selective destruction of p53-deficient (p53−) tumors has remained one of the most important goals and challenges of cancer therapy. One strategy for destroying p53− tumors is to inactivate genes that are preferentially required for the growth or survival of p53− cells. Here we carry out a large-scale genetic screen to identify a cellular pathway that is preferentially required for growth of p53− cancer cells.

## Introduction

The p53 tumor suppressor (also called TP53; NP_000537.3) plays a pivotal role in regulating multiple cellular processes including cell cycle arrest, apoptosis, cell metabolism and senescence (reviewed in [1]). Activated p53 can either induce cell cycle arrest and inhibit cell growth or promote cell apoptosis depending on the type of stress and the cellular context. Mutations that perturb p53 function, typically in its DNA-binding domain, or disruptions of the p53 upstream or downstream regulatory networks, have been found in more than half of all cancer cases and are present in cancer-prone families with Li-Fraumeni syndrome (OMIM#151623) (reviewed in [2]). In addition, loss of p53 function is often associated with increased resistance to chemotherapy and/or poor survival (see, for example, [3]–[5]). For these reasons, the selective molecular targeting of p53-deficient (p53−) tumors has remained one of the most important goals and challenges of molecular oncology (reviewed in [6]).

One strategy for treating p53− tumors is to reestablish the growth-inhibitory functions of p53. The feasibility of this approach is supported by animal studies demonstrating that reactivation of wild type p53 leads to tumor regression [7]–[9]. Several experimental strategies have been used to restore p53 activity. For example, gene therapy involving viral vectors has been used to reintroduce p53 into p53− tumor cells [10]. Alternatively, for cancers that retain a wild type *p53* gene, small molecule drugs have been identified that stabilize and activate p53 by interfering with its negative regulator MDM2 (NP_002383.2) (reviewed in [11]). Restoration of p53 function in cancers expressing only mutant p53 is even more challenging. However, small molecules that refold mutant p53 proteins, and thus reactivate p53 function, have been described (reviewed in [12]). Some of these approaches have progressed to clinical trials but to date none have been found to have clearly demonstrable clinical benefit [13].

An alternative approach to restoration of p53 function would be to target proteins that are preferentially required for proliferation or survival of p53− cells. Such targets can, in principle, be identified by synthetic lethal interaction screens, an idea first proposed by Hartwell et al. based upon studies in the budding yeast *Saccharomyces cerevisiae* [14]. The validity of this approach is supported by the realization that cancer cells are highly dependent upon or “addicted” to specific genes and regulatory pathways, confirming the existence of cancer cell-selective synthetic interaction targets (reviewed in [15], [16]). In addition, an important proof-of-principle is the demonstration that small molecule inhibitors of poly (ADP-ribose)-polymerase (NP_001609.2) are synthetically lethal with loss-of-function mutations in BRCA1 (NP_009225.1), BRCA2 (NP_000050.2) as well as other components of the homologous recombination pathway [17]–[19]. Here we carry out an RNA interference (RNAi)-based synthetic interaction screen to identify a regulatory pathway that is preferentially required for proliferation of p53− cancer cells.

## Results

### A Genome-Wide Short Hairpin RNA (shRNA)–Based Synthetic Interaction Screen Identifies Candidate Genes Preferentially Required for Proliferation of p53− Cells

**Figure 1:**
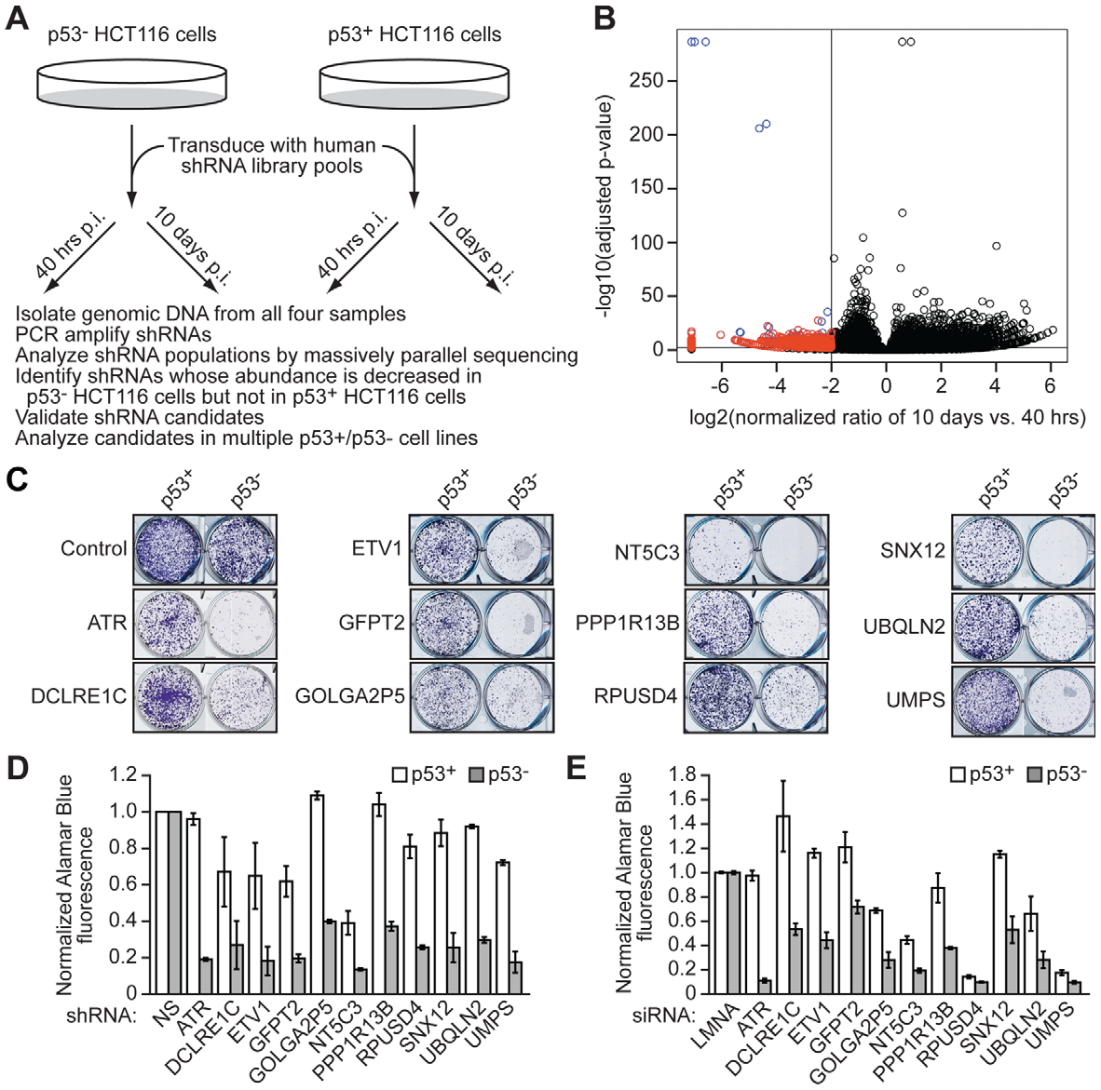
A genome-wide shRNA–based synthetic interaction screen identifies candidate genes preferentially required for proliferation of p53− cells. (A) Schematic summary of the screen. p53+ and p53− HCT116 cells were infected in parallel with a pooled lentiviral human shRNA library. The shRNA population was analyzed by massively parallel sequencing at 40 hours and 10 days post-infection (p.i.). (B) Volcano plot. The horizontal and vertical lines indicate the selection criteria. The red points represent shRNAs diminished ≥4-fold in p53− HCT116 cells and ≤2-fold in p53+ HCT116 cells at 10 days p.i. relative to 40 hours p.i. Blue points represent shRNAs diminished in both p53+ and p53− cells, and black points represent shRNAs not diminished in either p53+ or p53− cells. (C) Colony formation assay. p53+ and p53− HCT116 cells infected with a lentivirus expressing individual candidate shRNAs were selected with puromycin and plated in a serial dilution series in 6-well plates. Only one dilution set is shown. Colonies were fixed and stained with crystal violet. Control refers to the empty lentiviral vector, pGIPZ. (D) Proliferation assay. p53+ and p53− HCT116 cells infected with a lentivirus expressing each individual candidate shRNA, or as a control a non-silencing (NS) shRNA, were selected with puromycin and cell proliferation determined by an Alamar Blue fluorescence assay. The results were normalized to that obtained with a NS shRNA, which was set to 1. Error bars represent SD. (E) Proliferation of p53+ and p53− HCT116 cells transfected with an siRNA directed against an individual candidate gene, or a control lamin A/C (LMNA) siRNA, was determined by an Alamar Blue fluorescence assay. The results were normalized to that obtained with the control shRNA, which was set to 1. Error bars represent SD.

To identify genes that are preferentially required for the viability and proliferation of p53− cancer cells, we designed a synthetic interaction screen, which is summarized in Figure 1A and briefly described below. The primary screen was carried out using a well-characterized isogenic pair of human HCT116 colorectal cancer cell lines, one harboring wild type p53 (p53+ HCT116) and the other bearing a homozygous p53 deletion (p53− HCT116) [20]. For these and all other cell lines used in this study, the presence or absence of functional p53 was confirmed by monitoring expression of the p53 target gene, p21 (also called CDKN1A; NP_510867.1) (Figure S1). A human shRNA library comprising ∼60,000 shRNAs directed against ∼27,000 genes [21] was packaged into lentivirus particles, pooled and used to infect in parallel the two HCT116 cell lines. Ten days later, genomic DNA from both cell lines was isolated, and shRNAs were PCR amplified and subjected to massively parallel sequencing; as a reference, the starting shRNA population in both cell lines (taken 40 hours post-infection) was also analyzed.

Statistical analysis of the four shRNA populations identified shRNAs targeting 103 genes (Table S1) whose abundance was significantly decreased in p53− HCT116 cells (≥4-fold) but not in p53+ HCT116 cells (≤2-fold) at 10 days post-infection relative to the earlier time point (Figure 1B). Such shRNAs are presumably synthetic with the p53 deletion, thus rendering p53− cells harboring these shRNAs inviable or growth impaired, and leading to their relative under-representation in the p53− HCT116 population.

To validate candidates isolated from the primary screen, each shRNA was analyzed in an independent colony formation assay. p53+ and p53− HCT116 cells were infected with a lentivirus expressing a single candidate shRNA, and 10 days later cells were puromycin selected, re-plated at low density, and monitored for colony formation. This secondary screen revealed 11 genes that, when knocked down, substantially decreased colony formation in p53− HCT116 cells compared to p53+ HCT116 cells (Figure 1C). ShRNAs targeting these 11 genes also preferentially decreased the ability of p53− HCT116 cells to proliferate in culture (Figure 1D and summarized in Table S2). Quantitative RT-PCR (qRT-PCR) confirmed in all cases that expression of the target gene was decreased in the knockdown cell line (Figure S2A).

To rule out the possibility that growth inhibition was due to an off-target effect of the shRNAs, for each of the 11 genes we derived a short interfering RNA (siRNA) whose sequence was unrelated to the original shRNA used in the experiments described above. Figure 1E shows that each siRNA also preferentially decreased proliferation of p53− HCT116 cells compared to p53+ HCT116 cells. In all cases, qRT-PCR analysis confirmed that the siRNA decreased expression of the target gene (Figure S2B).

### Two Candidates, ETV1 and ATR, Are Preferentially Required for Proliferation of Diverse p53− Cell Lines

**Figure 2:**
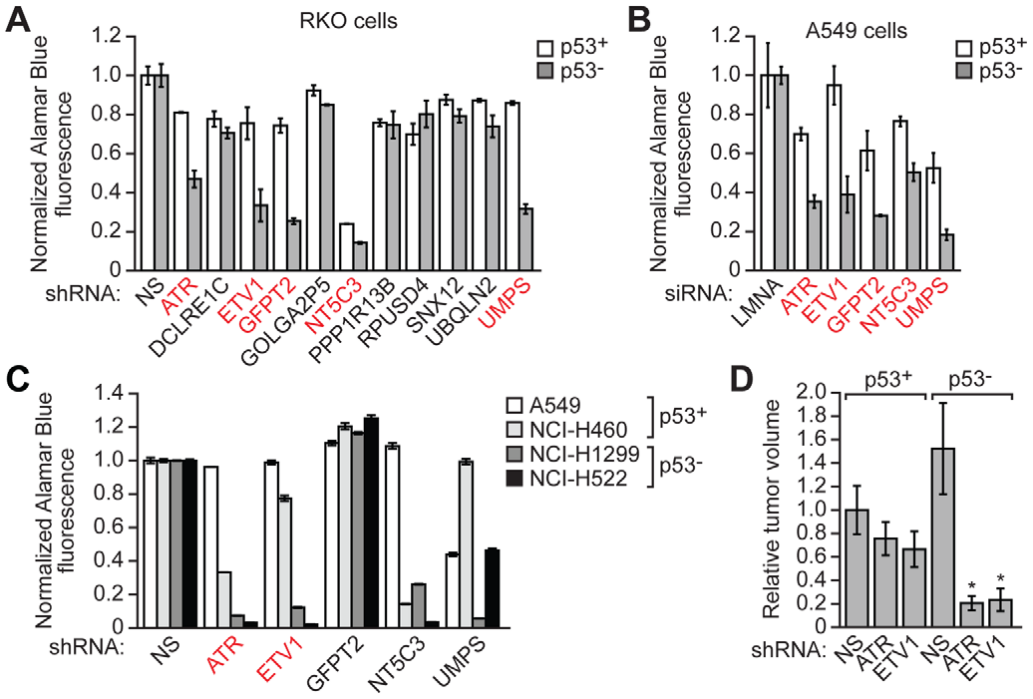
ETV1 and ATR are preferentially required for proliferation of diverse p53− cell lines. (A) Proliferation of p53+ and p53− RKO cells expressing an individual candidate shRNA, or as a control a NS shRNA, was determined by an Alamar Blue fluorescence assay. The results were normalized to that obtained with the NS shRNA, which was set to 1. Error bars represent SD. ShRNAs that preferentially affect the proliferation of p53− relative to p53+ cell lines are indicated in red. (B) Proliferation of p53+ and p53− A549 cells transfected with a candidate siRNA, or as a control a LMNA siRNA, was determined by an Alamar Blue fluorescence assay. The results were normalized to that obtained with the LMNA siRNA, which was set to 1. Error bars represent SD. ShRNAs that preferentially affect the proliferation of p53− relative to p53+ cell lines are indicated in red. (C) Proliferation of A549, NCI-H460, NCI-H1299 and NCI-H522 cells expressing a candidate shRNA, or as a control an NS shRNA, was determined by an Alamar Blue fluorescence assay. The results were normalized to that obtained with the NS shRNA, which was set to 1. Error bars represent SD. ShRNAs that preferentially affect the proliferation of p53− relative to p53+ cell lines are indicated in red. (D) p53+ and p53− HCT116 cells expressing an ATR or ETV1 shRNAs or as a control a NS shRNA, were subcutaneously injected into opposite flanks of the same nude mouse, and tumor volume was measured after 4 weeks. The results were normalized to that obtained in p53+ cells expressing a NS shRNA, which was set to 1. Error bars represent SD. * *P*≤0.0001.

p53− tumors from both the same and different types of cancers vary substantially with regard to additional genetic and epigenetic aberrations [22]. We were therefore interested in determining whether the 11 genes we identified were also preferentially required for the growth of other p53− cancer cells. To address this issue, we first analyzed an isogenic pair of human RKO colorectal cancer cell lines, one harboring wild type p53 (p53+ RKO cells) and the other bearing a homozygous p53 deletion (p53− RKO cells) (see Figure S1). ShRNAs to the 11 genes were introduced into the isogenic pair of RKO cell lines and proliferation was monitored. The results of Figure 2A indicate that five genes (*ATR* [NP_001175.2], *ETV1* [NP_001156619.1], *GFPT2* [NP_005101.1], *NT5C3* [NP_001002009.1] and *UMPS* [NP_000364.1]) were preferentially required for growth of p53− RKO cells compared to p53+ RKO cells. By contrast, knockdown of the other six genes did not substantially inhibit growth of either p53− or p53+ RKO cells and were thus not further analyzed.

We next examined these five candidates in an unrelated isogenic pair of A549 human lung cancer cell lines. In this case, the parental p53+ A549 cells were rendered p53− by stable expression of a p53 dominant-negative mutant [23] (see Figure S1). The results of Figure 2B show that siRNAs against the five candidate genes (*ATR*, *ETV1*, *GFPT2*, *NT5C3* and *UMPS*) preferentially inhibited growth of the p53− A549 cell line.

Finally, we analyzed the five candidate genes in a panel of four human lung cancer cell lines, two of which expressed wild type p53 (A549 and NCI-H460) and two of which were compromised for p53 function (NCI-H1299, which lacks p53, and NCI-H522, which expresses a p53 mutant) (see Figure S1). Of the five candidate genes, knockdown of two, *ATR* and *ETV1*, were the most consistent in preferentially inhibiting proliferation of p53− cell lines (Figure 2C) and were selected for further analysis. *ATR* encodes a checkpoint kinase involved in the DNA damage response [24], and *ETV1* encodes a member of the ETS family of transcription factors [25].

We also tested whether knockdown of *ATR* and *ETV1* would preferentially inhibit growth of p53− HCT116 tumors in a mouse xenograft model. p53+ or p53− HCT116 cells expressing an shRNA against ATR or ETV1, or a control non-silencing shRNA, were injected subcutaneously into opposite flanks of the same nude mouse, and tumor growth was monitored after four weeks. As expected, the control p53− HCT116 cells formed larger tumors than their p53+ counterparts (Figure 2D). Notably, knockdown of ATR or ETV1 markedly inhibited growth of p53− HCT116 tumors but did not have a significant effect on growth of p53+ HCT116 tumors.

### ETV1 and ATR Are Preferentially Required for *TERT* Expression in p53− Cells

**Figure 3:**
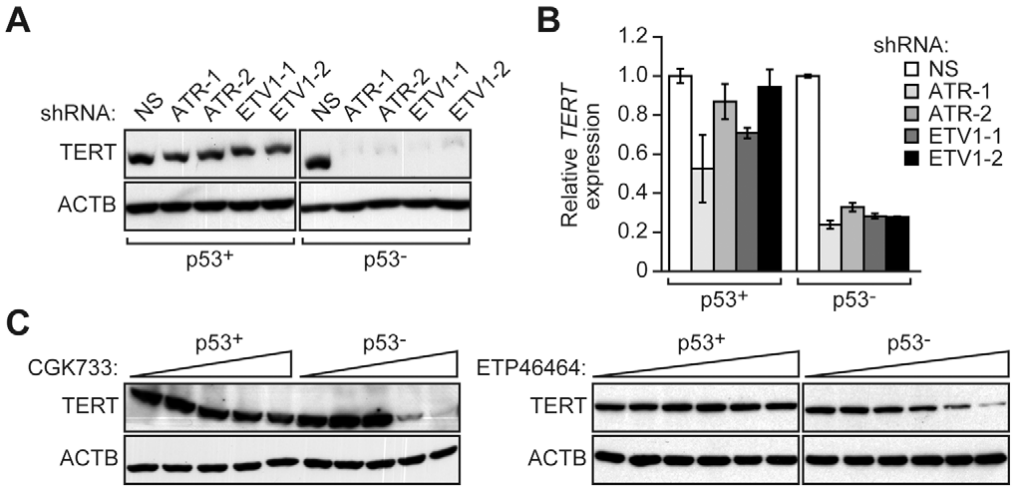
ETV1 and ATR are preferentially required for *TERT* expression in p53− cells. (A) Immunoblot analysis showing TERT levels in p53+ and p53− HCT116 cells expressing NS, ATR or ETV1 shRNAs. The results using two unrelated ATR or ETV1 shRNAs are shown. β-actin (ACTB) was monitoring as a loading control. (B) qRT-PCR analysis monitoring *TERT* expression in p53+ and p53− HCT116 cells expressing NS, ATR or ETV1 shRNAs. *TERT* expression was normalized to that obtained with a NS shRNA, which was set to 1. Error bars represent SD. (C) Immunoblot analysis showing TERT levels in p53+ and p53− HCT116 cells treated with the ATR inhibitor CGK733 (left; 0, 2, 3, 4, and 5 µM) or ETP46464 (right; 0, 0.5, 1, 2, 4 and 8 µM).

We next sought to investigate the basis by which ETV1 and ATR were preferentially required for growth of p53− cells. A previous study has shown that ETV1 is a transcriptional activator of *TERT* (NP_001180305.1) [26], which encodes the catalytic subunit of telomerase and has a well-established role in the maintenance of cellular proliferation [27]. Therefore, in the first set of experiments we analyzed the effect of depleting ETV1 as well as ATR on TERT levels. Significantly, RNAi-mediated knockdown of ETV1 or ATR resulted in a substantial decrease in TERT protein (Figure 3A) and mRNA (Figure 3B) levels in p53− HCT116 cells but unexpectedly had only a modest effect on TERT levels in p53+ HCT116 cells. The effect of knockdown of both ETV1 and ATR in p53− HCT116 cells on cellular proliferation and TERT levels was similar to that observed with single knockdowns (Figure S3A). Pharmacological inactivation of ATR using two different chemical inhibitors, CGK773 [28] and ETP46464 [29], also resulted in decreased TERT levels in p53− but not p53+ HCT116 cells (Figure 3C). Inhibition of ATR was confirmed by monitoring phosphorylation of its target substrate, CHK1 (also known as CHEK1; NP_001107593.1) (Figure S4).

**Figure 4:**
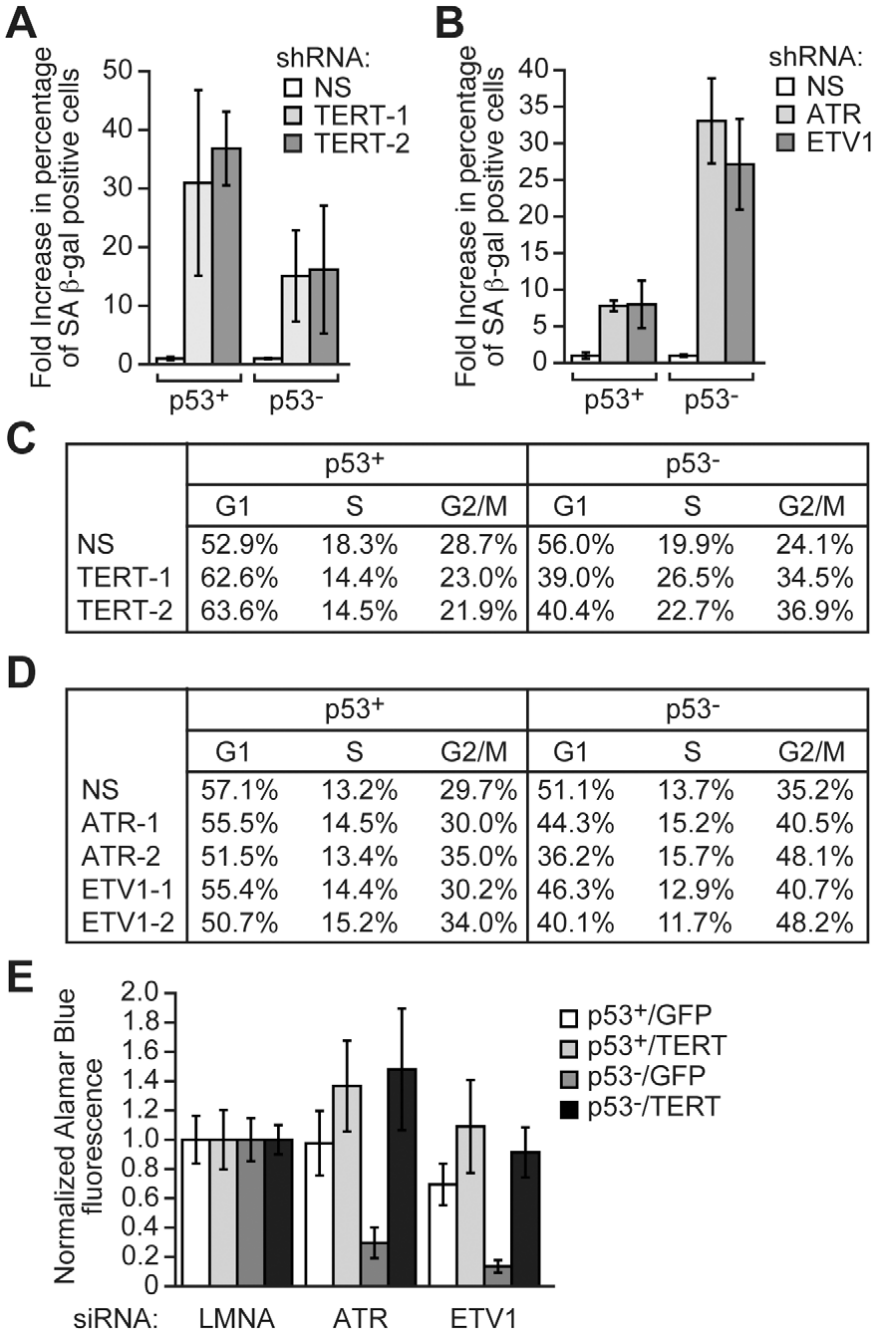
RNAi–mediated knockdown of ATR, ETV1, or TERT induces senescence and prolongs G2/M preferentially in p53− cells. (A) Senescence-associated β-galactosidase assay in p53+ and p53− HCT116 cells expressing a NS shRNA or one of two unrelated TERT shRNAs. Senescence-associated β-galactosidase activity was normalized to that obtained using a NS shRNA, which was set to 1. Error bars represent SD. (B) Senescence-associated β-galactosidase assay in p53+ and p53− HCT116 cells expressing a NS, ATR or ETV1 shRNA. Senescence-associated β-galactosidase activity was normalized to the level obtained using a NS shRNA, which was set to 1. Error bars represent SD. (C) Table showing the percentage of cells in G1, S and G2/M in p53+ and p53− HCT116 cells expressing a NS shRNA or one of two unrelated TERT shRNAs. (D) Table showing the percentage of cells in G1, S and G2/M in p53+ and p53− HCT116 cells expressing a NS shRNA or one of two unrelated ATR or ETV1 shRNAs. (E) Proliferation of p53+ and p53− HCT116 cells transfected with a control (LMNA), ATR or ETV1 siRNA and stably expressing TERT, or as a control GFP, was determined by an Alamar Blue fluorescence assay. Cell proliferation was normalized to that obtained using a LMNA siRNA, which was set to 1. Error bars represent SD.

We also monitored senescence induction and performed cell cycle analysis in cells depleted of ETV1 or ATR. Figure 4A shows that in both p53+ and p53− HCT116 cells, RNAi-mediated knockdown of TERT substantially increased the number of cells that stained positively for senescence-associated β-galactosidase activity, indicative of senescence induction (see also Figure S5A). The level of senescence was higher in p53+ HCT116 TERT knockdown cells than in p53− HCT116 TERT knockdown cells, as expected, because p53-directed pathways contribute to senescence [1]. Significantly, Figure 4B shows that RNAi-mediated knockdown of ETV1 or ATR also induced senescence (see also Figure S5B). However, following knockdown of ETV1 or ATR, the induction of senescence was much greater in p53− HCT116 cells compared to p53+ HCT116 cells (Figure 4B and Figure S5C), consistent with the difference in TERT levels (see Figure 3A). In addition, knockdown of TERT increased the percentage of p53− HCT116 cells but not p53+ HCT116 cells in G2/M (Figure 4C and Figure S6A). Notably, a similar preferential increase in the percentage of p53− HCT116 cells in G2/M occurred following knockdown of ETV1 or ATR (Figure 4D and Figure S6B, S6C).

To determine whether decreased TERT levels were responsible for the preferential growth defect in p53− HCT116 cells depleted of ETV1 or ATR, we performed a “rescue” experiment. Proliferation was measured both by an Alamar Blue assay (Figure 4E) and by quantifying cell number (Figure S7A) following knockdown of ETV1 or ATR in p53+ and p53− HCT116 cell lines stably expressing either TERT or, as a control, green fluorescence protein (GFP) (Figure S7B). The results of Figure 4E and Figure S7A show that ectopic expression of TERT counteracted the large decrease in proliferation that occurred following knockdown of ETV1 or ATR in p53− HCT116 cells, as well as the modest proliferative decrease following ETV1 knockdown in p53+ HCT116 cells. Thus, the reduced TERT levels following depletion of ETV1 or ATR can largely explain the decreased proliferation of p53− HCT116 cells. Consistent with this conclusion, TERT knockdown had a similar effect on proliferation of p53− cell lines to that observed following knockdown of ETV1 or ATR (see Figure S3). Moreover, knockdown of both ETV1 and TERT, or ATR and TERT, decreased proliferation of p53− cell lines similarly to that observed with single knockdowns (Figure S3).

### ATR Interacts with and Phosphorylates ETV1

**Figure 5:**
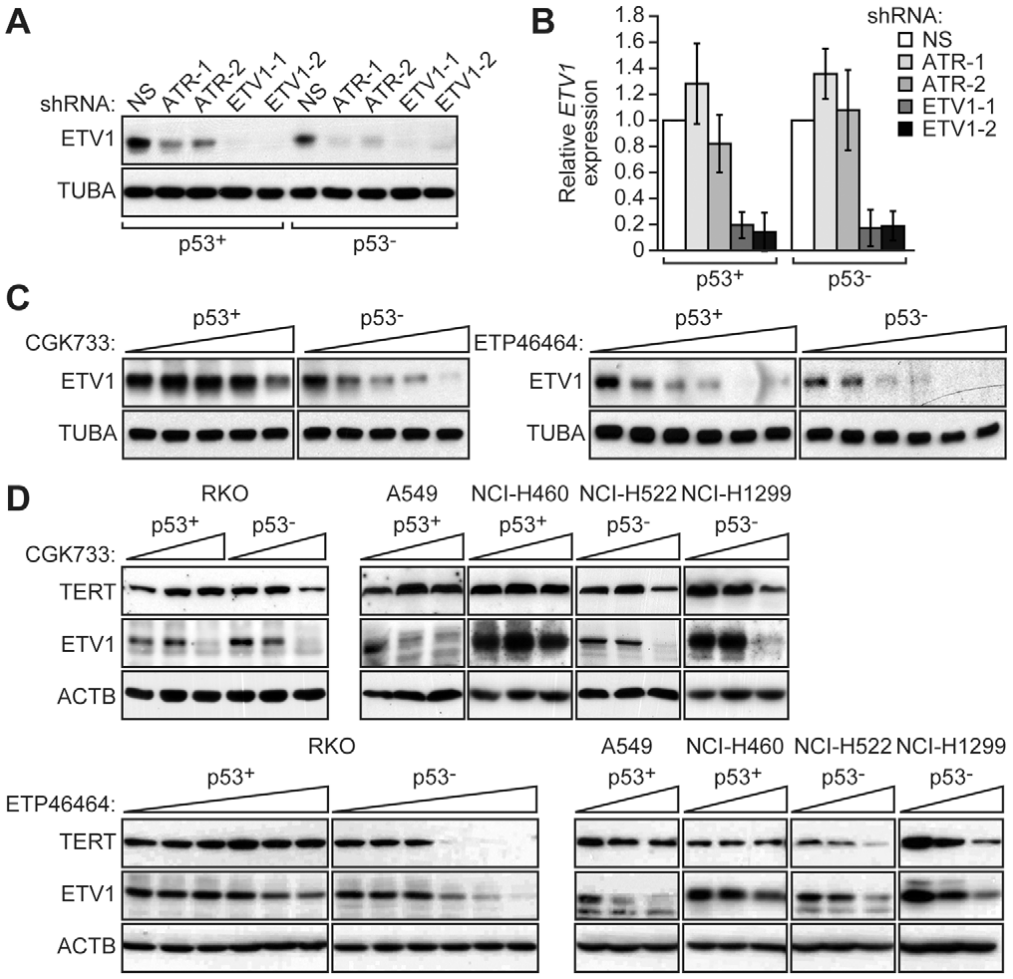
ATR is required for ETV1 stabilization. (A) Immunoblot analysis showing ETV1 levels in p53+ and p53− HCT116 cells expressing a NS, ATR or ETV1 shRNA. α-tubulin (TUBA) was monitoring as a loading control. (B) qRT-PCR analysis monitoring *ETV1* expression in p53+ and p53− HCT116 cells expressing a NS, ATR or ETV1 shRNA. *ETV1* expression was normalized to that obtained with a NS shRNA, which was set to 1. Error bars represent SD. (C) Immunoblot analysis showing ETV1 levels in p53+ and p53− HCT116 cells treated with CGK733 (left; 0, 2, 3, 4 and 5 µM) or ETP46464 (right; 0, 0.5, 1, 2, 4 and 8 µM). (D) Immunoblot analysis showing TERT and ETV1 levels in p53+ and p53− RKO cells, as well as A549, NCI-H460, NCI-H522 and NCI-H1299 cells treated with CGK733 (top; 0, 2 and 4 µM) or ETP46464 (bottom; 0, 0.5, 1, 2, 4 and 8 µM).

As described above, ETV1 has been previously shown to transcriptionally stimulate *TERT* expression [26]. However, the basis by which ATR promotes *TERT* expression is unknown. The similar results obtained with ATR and ETV1 in the *TERT* expression experiments of Figure 3 (and Figure S3) raised the possibility that the two proteins act in a common pathway. To address this possibility, we first asked whether ATR regulates ETV1 levels. The immunoblot results of Figure 5A show that in both p53+ and p53− HCT116 cells knockdown of ATR resulted in reduced ETV1 protein levels. The qRT-PCR results of Figure 5B showed that ATR depletion did not lead to reduced *ETV1* mRNA levels, indicating that the ATR-mediated regulation of ETV1 occurs post-transcriptionally. Addition of an ATR chemical inhibitor also led to reduced ETV1 protein levels in both p53+ and p53− HCT116 cells (Figure 5C). Following inhibition of ATR activity, DNA damage did not result in ETV1 stabilization (Figure S8).

To test the generality of these results, we analyzed the effect of ATR pharmacological inhibition in several of the p53+ and p53− cell lines described above. Figure 5D shows that ATR inhibition reduced TERT protein levels in p53− RKO, NCI-H522 and NCI-H1299 cells but not in p53+ RKO, A549 and NCI-H460 cells Moreover, addition of an ATR inhibitor reduced ETV1 levels to varying extents in all cell lines. TERT protein levels were also reduced following knockdown of ATR or ETV1 in NCI-H522 cells and two additional human cancer cell lines that express mutant p53 (Figure S9A), as well as in HeLa cells, which lack p53 activity due to expression of the human papilloma virus E6 protein (Figure S9B). Thus, the results in these other p53+ and p53− cell lines are similar to those obtained in the isogenic pair of HCT116 cell lines used throughout this study.

**Figure 6:**
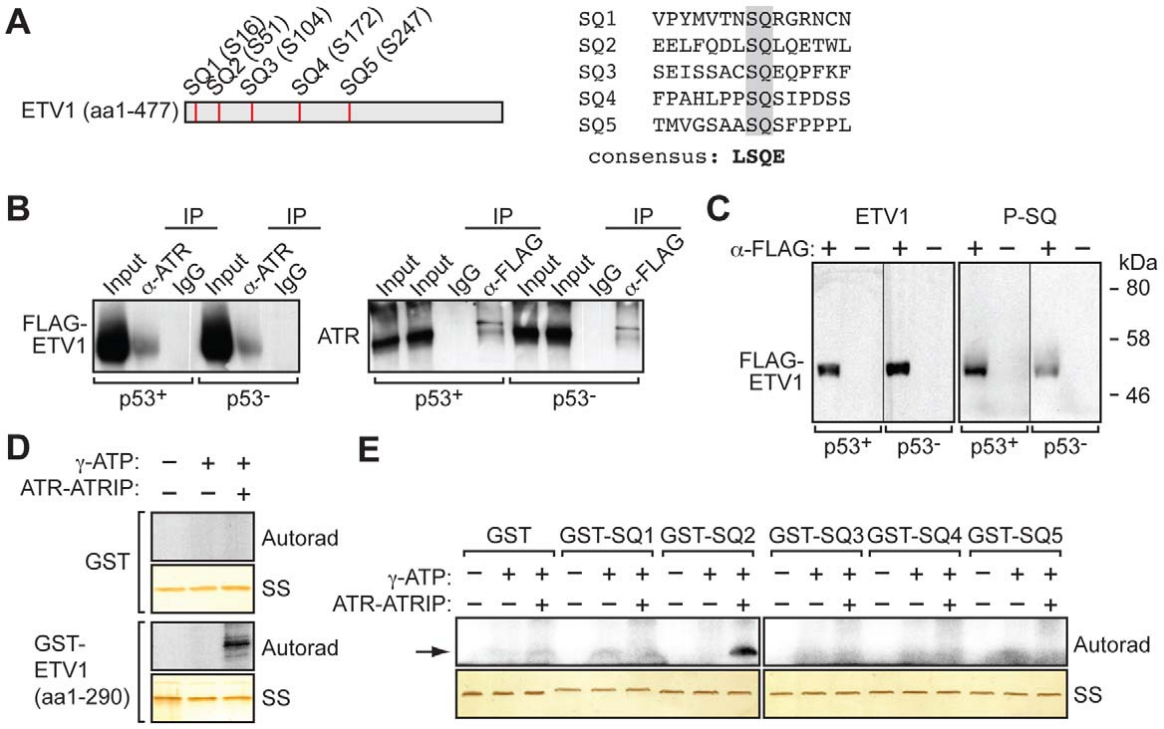
ATR interacts with and phosphorylates ETV1. (A) (Left) Schematic of the full-length ETV protein, showing the positions of the five potential ATR phosphorylation sites (SQ). (Right) Sequence surrounding each potential phosphorylation site, and the consensus ATR phosphorylation site. (B) Co-immunoprecipitation assay. Cell extract from p53+ or p53− HCT116 cells expressing FLAG-ETV1 was immunoprecipitated using an ATR antibody and the immunoprecipitate analyzed by immunoblotting for FLAG (left), or immunoprecipitated using a FLAG antibody and the immunoprecipitate analyzed by immunoblotting for ATR (right). IgG was used as a specificity control. (C) Extract from p53+ or p53− HCT116 cells expressing FLAG-ETV1 was immunoprecipitated using a FLAG antibody and the immunoprecipitate analyzed by immunoblotting using an antibody that recognizes ETV1 or a phosphorylated SQ motif (P-SQ). (D) In vitro kinase assay monitoring the ability of ATR to phosphorylate a GST-ETV1 (amino acids 1–290) fusion protein containing all five potential SQ phosphorylation sites or, as a control, GST alone. Autoradiographic images (Autorad, top) and corresponding silver-stained gels (SS, bottom) are shown. (E) In vitro kinase assay monitoring the ability of ATR to phosphorylate a series of GST-ETV1 fusion proteins, each containing 15 amino acids encompassing a potential SQ phosphorylation site (sequences shown in A) or, as a control, GST alone. Autoradiographic images (Autorad, top) and corresponding silver-stained gels (SS, bottom) are shown. The position of the ^32^P-labeled fusion protein is indicated by the arrow.

Because ATR is a protein kinase, a likely mechanism for the ability of ATR to post-transcriptionally regulate ETV1 is through direct interaction and phosphorylation. Consistent with this possibility, ETV1 contains five potential ATR phosphorylation sites (Figure 6A). To test this idea, we ectopically expressed a FLAG-tagged ETV1 derivative (Figure S10) in p53+ and p53− HCT116 cells, and analyzed interaction between FLAG-ETV1 and ATR in a co-immunoprecipitation assay. The results of Figure 6B show that in both p53+ and p53− HCT116 cells, FLAG-ETV1 could be detected in the ATR immunoprecipitate (left) and, conversely, ATR could be detected in the FLAG immunoprecipitate (right), indicating ATR and ETV1 physically associate. To determine whether ETV1 was an ATR substrate, we immunoprecipitated FLAG-ETV1 from transfected p53+ and p53− HCT116 cell lysates and analyzed the immunoprecipitate by immunoblotting with an antibody that recognizes a phosphorylated serine followed by a glutamine [30], the product of ATR or ATM phosphorylation [31], [32]. The results of Figure 6C show that the immunoprecipitated FLAG-tagged ETV1 could be detected by the ATM/ATR phospho-specific antibody, suggestive of phosphorylation by ATR. Moreover, following treatment of cells with an ATR inhibitor, the immunoprecipitated FLAG-tagged ETV1 was no longer detected by the ATM/ATR phospho-specific antibody (Figure S11).

To confirm that ATR phosphorylates ETV1, we performed in vitro kinase experiments. We first tested whether ATR, in the presence of its positive effector ATRIP (NP_569055.1) [33], [34], could phosphorylate a glutathione-S-transferase (GST)-ETV1 (amino acids 1–290) fusion-protein that contained all five potential ATR phosphorylation sites. The results of Figure 6D show that ATR phosphorylated the GST-ETV1 fusion-protein but, as expected, not a control GST protein. To confirm and extend this result, we constructed and analyzed a series of GST-ETV1 fusion-proteins each containing a single potential ATR phosphorylation site. The results of Figure 6E show that only one of the five potential ATR phosphorylation sites (SQ2) was a substrate for ATR. Collectively, the results described above indicate that ATR phosphorylates ETV1 and stabilizes it from proteolytic degradation.

### ETV1 and ATR Are Bound to the *TERT* Promoter in p53− but Not p53+ Cells

**Figure 7:**
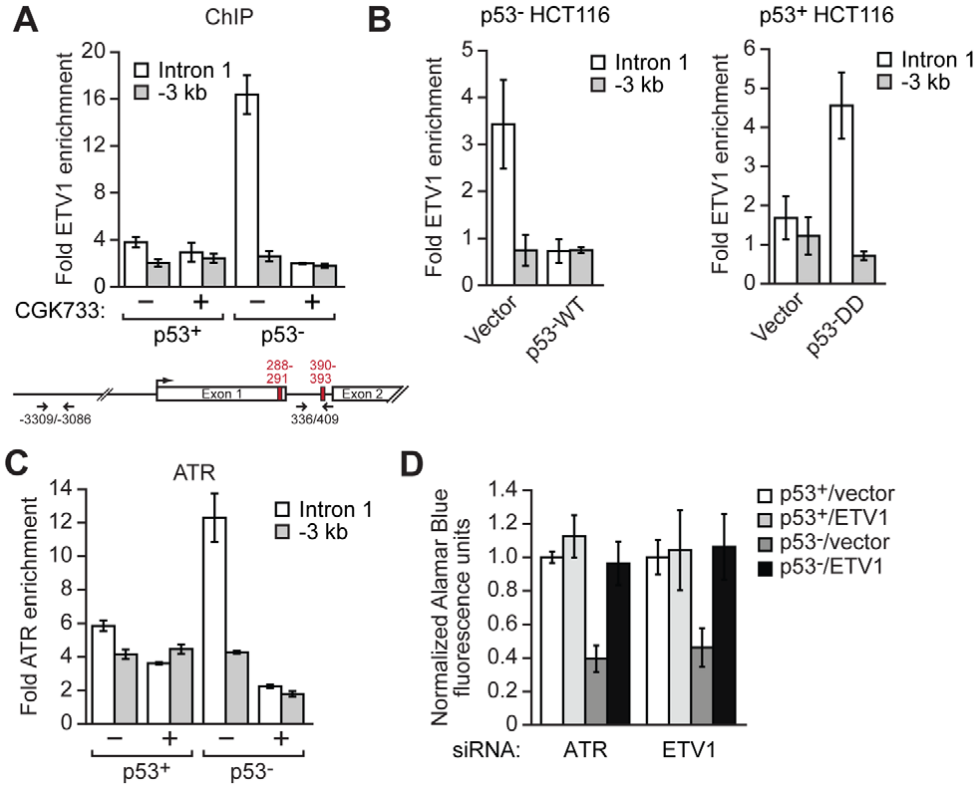
ATR and ETV1 are bound to the *TERT* promoter in p53− but not p53+ cells. (A) ChIP analysis monitoring ETV1 occupancy at two regions of the *TERT* promoter, in the first intron or 3 kb upstream of the transcription start-site, in p53+ and p53− HCT116 cells treated in the presence or absence of CGK733. The locations of the primer pairs (arrows) and ETV1-binding sites (red rectangles) are shown in the schematic of the *TERT* promoter (bottom). Error bars represent SD. (B) ChIP analysis monitoring ETV1 occupancy at two regions of the *TERT* promoter in p53− HCT116 cells expressing wild type p53 (p53-WT) or a vector control (left) and in p53+ HCT116 cells expressing a dominant negative p53 mutant (p53-DD) or a vector control (right). Error bars represent SD. (C) ChIP analysis monitoring ATR occupancy at two regions of the *TERT* promoter in p53+ and p53− HCT116 cells treated in the presence or absence of CGK733. Error bars represent SD. (D) Proliferation of p53+ and p53− HCT116 cells stably expressing ETV1 or, as a control, empty vector was determined by an Alamar Blue fluorescence assay. Proliferation was normalized to that obtained using a LMNA siRNA, which was set to 1 (not shown). Error bars represent SD.

As discussed above, previous studies have shown that ETV1 is a transcriptional activator of *TERT* [26]. Therefore, we thought the most likely mechanism by which ETV1 promotes proliferation in p53− HCT116 cells is through direct binding to the *TERT* promoter and stimulation of *TERT* transcription. To test this possibility, we performed chromatin-immunoprecipitation (ChIP) experiments. The ChIP experiments of Figure 7A (left panel) show that in p53− HCT116 cells, ETV1 was bound to a region within intron 1, which has been previously reported to contain multiple ETV1 binding sites and is required for complete *TERT* transcriptional activity [26]. Remarkably, in p53+ HCT116 cells, whose proliferation is not dependent upon ETV1, there was no detectable binding of ETV1 to the same region of the *TERT* promoter. Notably, ectopic expression of wild type p53 in p53− HCT116 cells resulted in substantially decreased binding of ETV1 to the *TERT* promoter (Figure 7B, left). Conversely, ectopic expression of a p53 dominant-negative mutant in p53+ HCT116 cells resulted in substantially increased binding of ETV1 to the *TERT* promoter (Figure 7B, right).

In p53− HCT116 cells, binding of ETV1 to the *TERT* promoter was lost following pharmacological inhibition of ATR (Figure 7A and Figure S12A), which as shown above results in decreased ETV1 levels (see Figure 5C). Conversely, binding of ETV1 to the *TERT* promoter modestly increased following irradiation with ultraviolet light, which increases ATR activity (Figure S12B). ChIP experiments monitoring ATR occupancy revealed that ATR was bound to the same region of the *TERT* promoter as ETV1 (Figure 7C). Thus, in p53− HCT116 cells, ETV1 and ATR are both bound to the *TERT* promoter, which is consistent with our finding that the two proteins are physically associated (Figure 6B).

In conjunction with a previous study [26], the results presented above suggested that ETV1 is directly responsible for stimulating *TERT* expression and that ATR functions by phosphorylating and thereby stabilizing ETV1. A prediction of this model is that ectopic expression of ETV1 would bypass the requirement of ATR for proliferation of p53− HCT116 cells. The rescue experiment of Figure 7D shows that the decreased proliferation of p53− HCT116 cells following knockdown of ATR was counteracted by ectopic expression of ETV1 (Figure S13). Following knockdown of TERT, ectopic expression of ETV1 could no longer rescue proliferation of p53− HCT116 cells depleted of ATR (Figure S14A). In these experiments, ectopic expression of ETV1 had no effect on γ-H2AX foci formation, a marker of DNA damage [35] (Figure S14B). These results suggest that the growth arrest observed following loss of ATR is primarily due to decreased ETV1 levels.

## Discussion

In this report we have performed a large-scale shRNA screen to identify a regulatory pathway involving ETV1, ATR and TERT that is preferentially required for proliferation of diverse p53− cancer cells. We found that in p53− cells, *TERT* transcription is highly dependent upon ETV1, which functions as a direct transcriptional activator by binding to the *TERT* promoter downstream of the transcription start-site. In p53+ cells, ETV1, although present at comparable levels, is not required for *TERT* transcription and surprisingly is not bound to the same region of the *TERT* promoter. Notably, ectopic *TERT* expression restored normal proliferation in p53− cells depleted of ETV1 or ATR (Figure 4E and Figure S7A), indicating that the promotion of *TERT* expression is an important, but not necessarily the only, mechanism by which ETV1 and ATR maintain proliferation of p53− cells. Consistent with our results, a previous study reporting a requirement for ETV1 in *TERT* transcription [26] was primarily based upon experiments performed in 293T cells, which lack p53 activity due to expression of SV40 large T antigen.

The results described above suggest that p53+ cells express a transcription factor that functionally substitutes for ETV1, and that one or more proteins associated with the *TERT* promoter in p53+ cells prevent binding of ETV1. Several transcription factors, including SP1 (NP_612482.2), E2F1 (NP_005216.1) and MYC (NP_002458.2), have been previously shown to be associated with the human *TERT* promoter (reviewed in [36]). To ask whether these factors, or p53 itself, might contribute to the differential regulation of TERT we performed ChIP experiments in p53+ and p53− HCT116 cells. Consistent with previous studies, we found that E2F1 and MYC were associated with the *TERT* promoter; binding of E2F1 was modestly increased in p53− HCT116 cells (Figure S15A), whereas for MYC there was no difference in p53+ and p53− HCT116 cells (Figure S15B). In p53+ HCT116 cells there was increased binding of SP1 (Figure S15C) and, most notably, there was substantial binding of p53 to the *TERT* promoter (Figure S15D). Interestingly, a number of previous studies have reported physical and functional interactions between SP1 and p53 (see, for example, [37]–[41]). Our ChIP results reveal substantial differences between the composition of proteins associated with the *TERT* promoter in p53+ and p53− HCT116 cells, which may be related to the differential requirement for ETV1.

Interestingly, in contrast to human cancer cell lines, we found that ATR was not required for *TERT* expression in experimentally derived p53− MCF10A cells, an immortalized but non-transformed human cell line (Figure S16A). In addition, ATR was not required for *TERT* expression in p53− mouse embryo fibroblasts (Figure S16B), consistent with the lack of conservation between the mouse and human *TERT* promoter (data not shown). Thus, the requirement of ATR and ETV1 for *TERT* expression may be specific to human p53− cancer cell lines.

Several previous studies have reported results that are consistent with the synthetic interaction between p53 and ATR we have described here. For example, p53− cells have been found to be particularly sensitive to pharmacological inhibition of ATR (see, for example, [42]–[44]). In addition, mice expressing a hypomorphic allele of *ATR* have an aging phenotype that is exacerbated in the absence of p53 [45]. Significantly, mouse embryo fibroblasts containing this hypomorphic *ATR* allele have an elongated G2 phase following loss of p53, consistent with our cell cycle results (Figure 4D and Figure S6B, S6C). However, a preferential role for ETV1 in p53− cells and its cooperative function with ATR has not been previously described and underscores the power of unbiased, large-scale RNAi-based screens.

Our screening strategy did not emphasize reaching saturation but rather sought to follow-up, by directed experiments, a limited number of candidates isolated in the primary screen. For several reasons, we believe that our screen, like other large-scale shRNA screens (see, for example, [46]), did not achieve saturation. For example, a previous siRNA screen identified several factors, in particular the serine/threonine kinase receptor-associated protein UNRIP (also called STRAP; NP_009109.3), whose loss affected proliferation of p53− HCT116 cells more severely than p53+ HCT116 cells [47]. However, we did not isolate UNRIP in our primary screen and, conversely, ATR and ETV1 were not isolated in the previous siRNA screen, suggesting that neither screen was truly saturating. Reasons for a failure to reach saturation in this and other large-scale shRNA screens include suboptimal efficacy of some shRNAs [48], unequal representation of shRNAs in the primary screen, and an insufficient depth of deep sequencing. Thus, it is possible that additional factors that act in the ATR-ETV1-TERT pathway, or unrelated pathways preferentially required for proliferation of p53− cells, remain to be identified.

The decreased proliferation of p53− cell lines was first evident within a few days following knockdown of ETV1, ATR or TERT. It therefore seems likely that this reduced proliferation is not a result of replicative senescence due to telomere attrition, which would require many cell divisions. Senescence occurred at much later times (10–14 days) and may be a secondary effect of the proliferation block. We observed that knockdown of ETV1, ATR or TERT resulted in an increased percentage of cells in G2/M (Figure 4C, 4D and Figure S6). Although senescent cells are generally believed to arrest in G1, it has been found that senescent cells can also arrest in G2/M (see, for example, [49]).

A variety of previous studies have shown that TERT can promote proliferation by multiple mechanisms, several of which are unrelated to telomere length including inhibiting apoptosis [50], regulating cell signaling pathways and/or stimulating expression of diverse growth-promoting genes (see, for example, [51]–[54]). It seems likely that the decreased proliferation of p53− cells following depletion of ETV1, ATR or TERT involves one of these alternative mechanisms. We have found that p53− cells depleted of ETV1, ATR or TERT have multiple growth defects including increased levels of senescence (Figure 4A, 4B and Figure S5) and an altered cell cycle (Figure 4C, 4D and Figure S6). A further understanding of how TERT promotes proliferation of p53− cells is likely to identify new factors that are potential therapeutic targets.

## Materials and Methods

### Ethics Statement

Animal experiments were performed in accordance with the Institutional Animal Care and Use Committee (IACUC) guidelines.

### Cell Lines and Culture

Isogenic p53+ and p53− HCT116 and RKO cell lines [20] were provided by B. Vogelstein; A549, NCI-H460, NCI-H522, NCI-H1299 and HT29 cells were obtained from the National Cancer Institute; and DLD-1, HeLa and MCF10A cells were obtained from the American Type Culture Collection. The basis for the p53− status in each of the p53− cell lines is provided in Table S3. p53+ and p53− mouse embryonic fibroblasts were isolated from wild type and p53^−/−^ C57BL/6 mice. All cells were grown according to the supplier's recommendations. Stable A549 and MCF10A cell lines expressing p53-DD, which harbors a deletion of 288 amino acids (Δ15-301; [23]) were generated by transfection with the plasmid pBABE-hygro-p53DD (Addgene; [55]) or the control vector, pBABE-hygro, and selection with hygromycin (150–200 µg/ml). Stable p53+ and p53− HCT116 cell lines expressing TERT were generated by transfection with the plasmid pWZL-Blast-Flag-HA-hTERT (Addgene; [56]) or control plasmid pWZL-Blast-GFP (Addgene; [57]), and selection with blasticidin (10 µg/ml). The ETV1 expression vector was generated by subcloning ETV1 cDNA (Open Biosystems) into pEF6-Blast-3xFlag to create pEF6-Blast-3xFlag-ETV1. The pEF6-Blast-3xFlag vector was generated by cloning a BsiWI-EcoRI double-stranded oligo coding for 3xFlag-tag (MDYKDHDGDYKDHDIDYKDDDDKEF) in Kpn1-EcoR1-digested pEF6/V5-HIS B (Invitrogen). Stable p53+ and p53− HCT116 cell lines expressing ETV1 were generated by transfection with pEF6-Blast-3xFlag-ETV1 or vector only and selection with blasticidin (10 µg/ml).

### RNAi Screening

The Open Biosystems GIPZ lentiviral human shRNAmir library was obtained through the University of Massachusetts Medical School RNAi Core Facility. Twelve lentiviral pools, each comprising ∼5000 shRNA clones, were generated with titers of ∼2×10^6^ pfu/ml. These lentiviral stocks were produced following co-transfection with the packaging mix into the 293T packaging cell line. To carry out the screen, p53+ and p53− HCT116 cells were plated at 1×10^6^ cells per 100 mm plate, transduced the next day with one shRNA pool per plate at a multiplicity of infection (MOI) of 1, and grown in the absence of puromycin selection. Forty hours after transduction, 75% of cells were transduced (as evidenced by GFP fluorescence; the marker turboGFP is present in the pGIPZ vector). Each plate was divided into two populations: half of the cells were pooled and genomic DNA was extracted (referred to as “T0”), whereas the other half were transferred to 150 mm plates and passaged by 4-fold dilutions for 10 days, at which point the cells were pooled and the genomic DNA was extracted (referred to as “T10”).

### Deep Sequencing

To analyze the frequency of individual shRNAs in the four populations, 72 µg of genomic DNA was used as the substrate (split into 24 tubes) and PCR amplified (94°C for 1 min; 15 cycles of 94°C for 1 min, 58°C for 1 min, 72°C for 45 sec; 72°C for 10 min; and hold at 4°C) with primers GIPZF (5′-GAGTTTGTTTGAATGAGGCTTCAGTAC-3′) and GIPZHR (5′-CGCGTCCTAG GTAATACGAC-3′). The PCR product was gel purified, and 50 ng of DNA was used as the substrate for a second PCR amplification (94°C for 1 min; 15 cycles of 94°C for 1 min, 50°C for 1 min, 72°C for 45 sec; 72°C for 10 min; and hold at 4°C) using primers Forward Acu1 primer AMN (5′-CAACAGAAGGCTCCTGAAGGTATATTGCTGTTGAC-3′) and Reverse Acu1 primer AMN (5′-AAATTTAAACTGAAGTACATCTGTGGCTTCACTA-3′). Next, 1 µg of the PCR product was digested to completion with AcuI (New England Biolabs). The digested product was then ligated to the following pre-annealed adapters: L1ShSolexA (/5Bio/-ACACTC TTTCCCTACACGACGCTCTTCCGATCTCA) and L1ShSolexB (/5Phos/′-AGATCGGAAGA GCGTCGTGTAGGGAAAGAGTGT/3AmM, and L2ShSolexB (/5Phos/-AGATCGGAAGAGC TCGTATGCCGTCTTCTGCTTG/3Bio/) and L2ShSolexA (/5AmMC6/-CAAGCAGAAGACG GCATACGAGCTCTTCCGATCTAC). The product of the 3-way ligation was run on a 3% TAE agarose gel, visualized with ethidium bromide, purified and used as a substrate for a 15-cycle PCR reaction using Solexa-Illumina primers 1.1 and 2.1 and the cycling conditions recommended by the manufacturer.

The library was analyzed using the Solexa-Illumina GA Massively Parallel Deep Sequencer. Sequence information was extracted from the image files using the Solexa-Illumina Firecrest and Bustard applications. Prior to alignment of the sequence reads, a custom Perl script was used to identify the first six bases flanking the informative sequence in 5′ and the six bases flanking the informative sequence in 3′, starting at position 28. The core 21 bp sequences were extracted and mapped to the human reference genome sequence (hg18) using the Solexa-Illumina ELAND algorithm, allowing up to two mismatches to the reference sequence. No further analysis was performed on reads that did not contain the six bases of the 5′ sequence or the six bases of 3′ adapter sequence.

Sequences mapping to the same genomic location were binned and the count for each of the mapped genomic sequences was calculated for each of the four treatments. For each of the mapped genomic sequences, the Fisher Exact Test was applied to assess whether there was a differential depletion/enrichment of the shRNA sequences between T0 and T10 for both the p53− and p53+ HCT116 cell lines. The odds ratio and its 95% confidence interval were computed for each of the mapped genomic sequences using Fisher test function in R v2.8 based on conditional maximum likelihood estimation. To adjust for multiplicity, B–H method [58] was used. Those shRNAs with an adjusted p-value<0.01 and a decrease of at least four-fold at T10 compared with T0 in p53− HCT116 cells and no more than two-fold in p53+ HCT116 (or adjusted p-value≥0.01) were identified. The data discussed in this publication have been deposited in NCBI's Gene Expression Omnibus [59] and are accessible through GEO Series accession number GSE15967 (http://www.ncbi.nlm.nih.gov/geo/query/acc.cgi?acc=GSE15967).

### Colony Formation Assay

Lentiviral supernatants corresponding to individual shRNAs (listed in Table S4) were generated in 293T cells as described above. p53+ and p53− HCT116 cells were transduced with each lentiviral preparation at an MOI of 0.2–0.4, and grown for 10 days without puromycin selection, during which cells were passaged at a 1∶6 ratio every 4 days. Cells were then subjected to puromycin selection (1.5 µg/ml) for 5 days. For colony formation assays, cells were split at a 1∶200 ratio and plated in 6-well plates in the presence of 1.5 µg/ml puromycin. After 6–7 days, cells were fixed with 4% paraformaldehyde in phosphate buffered saline (PBS) at 4°C overnight and then stained with 0.1% crystal violet in PBS to visualize the colonies. At least two independent infections were performed for each shRNA; representative images are shown.

### Alamar Blue Assays

For shRNA-based experiments, cells were transduced with lentiviral supernatants at an MOI of 0.2–0.4, and subjected to puromycin selection (1.5 µg/ml) for 5 days. Cells were then passaged at a 1∶8 ratio every 3 days and cultured in growth medium containing puromycin. After 4 passages, cells were split at a 1∶6 ratio and seeded in a 12-well plate in RPMI medium without phenol red and supplemented with 5% fetal calf serum. After 18 h, the medium was replaced with 500 µl of medium containing 10% of an Alamar Blue solution (Invitrogen). After 2 h, 100 µl of the medium was used to measure fluorescence by excitation at 530 nm and emission at 590 nm. For siRNA-based experiments, siRNA duplexes were transfected into cells using Lipofectamine RNAiMax Transfection Reagent (Invitrogen) according to the manufacturer's instructions. Briefly, 1.2 µl of Lipofectamine was complexed with the siRNA (40 nM final concentration), and the solution was diluted with 100 µl of medium and applied to 2×10^4^ cells in a volume of 500 µl culture medium per well in 24-well plates. The medium was changed after 24 h and proliferation assessed by Alamar Blue fluorescence after an additional 72 h. Sequences of siRNAs are listed in Table S4; the control LMNA siRNA sequence was previously described [60]. All experiments were performed at least 2–3 times in either duplicate or triplicate.

### Trypan Blue Cell Counting

Four days post-siRNA-transfection, cells were trypsinized, resuspended in 0.5 ml growth medium, and stained with 0.5 ml 0.1% Trypan blue solution (HyClone Trypan blue, Thermo Fisher Scientific). Viable cells were counted using a Countess Automated Cell Counter (Life Technologies). Two independent transfections were carried out and analyzed in duplicate.

### Tumor Formation Assays

2×10^6^ shRNA-transduced p53+ or p53− HCT116 cells were suspended in 100 µl of serum-free RPMI and injected subcutaneously into the opposite flanks of n = 9 (for non-silencing and ATR shRNAs) or n = 5 (for ETV1 shRNA) athymic Balb/c (nu/nu) mice (Taconic). Tumor dimensions were measured every week and tumor volume was calculated using the formula π/6×(length)×(width)^2^. A Mann-Whitney test was used to determine whether knockdown of ATR or ETV1 changes the tumor volume at week 4 compared to a non-silencing shRNA.

### Antibodies and Immunoblot Analysis

Cell extracts were prepared by lysis in modified RIPA buffer (0.05 M Tris-Cl [pH 8.0], 0.15 M NaCl, 1% Nonidet P-40, 0.5% desoxycholate, 0.1% SDS, 2 mM phenylmethylsulphonyl fluoride (PMSF), 20 µg/ml aprotinin, 1 mM Na3VO4 and 1 mM NaF) in the presence of a proteinase inhibitor cocktail (Roche). Blots were probed with α-TERT (Epitomics, 1531-1). α-ETV1 (Abcam, ab81086), α-Flag M2 (Sigma, F1804), α-phospho-CHK1(Ser317) (Cell Signaling Technology, 8191), α-p21 (BD Pharmingen, SX118), α-tubulin (Sigma, B5-1-2) or β-actin (Sigma, AC74). For ATR inhibition, cells were treated with 2–6 µM CGK733 (Calbichem) or 0.5–8 µM ETP46464 ([29]; kindly provided by O. Fernandez Capetillo) for 72 h prior to cell extract preparation; as a control, cells were treated with dimethyl sulfoxide (DMSO). For p53 functional assays (Figure S1), cells were treated with 25 µM etoposide (Sigma) or 10 µg/ml 5-fluorouracil (Sigma) for 24 h, and cell extracts were prepared as above. For RNAi experiments, experiments were performed at least 2–3 times in either duplicate or triplicate.

### qRT–PCR

Total RNA was extracted using TRIzol Reagent (Invitrogen) and treated with Turbo DNA-free kit (Ambion Inc.). The same amount of total RNA (3 µg) for each sample was employed to produce templates for SYBR-green quantitative PCR analysis using SuperScript II Reverse transcriptase (Invitrogen). Target genes were amplified using specific primers and expression levels were normalized to that of GAPDH. Primer sequences are listed in Table S4. All experiments were performed at least 2–3 times in either duplicate or triplicate.

### Senescence-Associated β-Galactosidase Assays

Assays were performed as described previously [61] with minor modifications. Briefly, 10–14 days following RNAi-mediated knockdown, cells were washed twice with PBS, then fixed using 3.7% paraformaldehyde for 5 min at room temperature. After three washes with PBS, cells were incubated with fresh staining solution (40 mM citric acid/Na_2_HPO_4_ pH 6.0, 150 mM NaCl, 2 mM MgCl_2_, 5 mM potassium ferricyanide, 5 mM potassium ferrocyanide, 1 mg/ml X-Gal) for 12–18 hr at 37°C (no CO_2_) and covered from light. Images were captured using a Spot TE-200 digital camera (SPOT Imaging Solutions). The number of blue cells in 10 fields (each containing 100–250 cells) was counted manually, and the percentage calculated. Two independent infections were performed for each knockdown.

### Cell Cycle Analysis

Cells transduced with shRNAs were harvested by trypsinization, fixed in 80% ethanol and stored at −20°C overnight. Fixed cells were stained with propidium iodide buffer containing 50 µg/ml RNase (Sigma) and 50 µg/ml propidium iodide (Sigma) in PBS. Flow cytometry was performed by the UMass Medical School Core Flow Cytometry Lab using a FACScalibur flow cytometer (Becton Dickinson). Data were analyzed with FlowJo (Tree Star). All experiments were performed at least 2–3 times.

### Co-Immunoprecipitation Assays

For Figure 6B, 5×10^7^ p53+ or p53− HCT116 cells expressing Flag-ETV1 were rinsed twice with cold PBS, lysed in 1 ml IP lysis buffer (50 mM Tris-Cl pH 7.4, 250 mM NaCl, 5 mM EDTA, 0.2%Triton X-100, 0.5 mM DTT, 1× complete protease inhibitor [Roche], and phosphatase inhibitor cocktails 2 and 3 [Sigma, p5726 and p0044]) on ice. The lysate was cleared by centrifugation at 16,000 g for 30 min at 4°C. Whole cell lysate (2 mg per sample) was incubated with relevant antibodies (α-ATR [Abcam, ab2905] or control rabbit IgG [Abcam, ab37415] or α-Flag M2 [Sigma] or control mouse IgG [Santa Cruz, sc2343]) overnight at 4°C after being precleared with 50 µl Dynabeads-protein G (Invitrogen). Dynabeads Protein G (50 µl) were added to each lysate-antibody complex, incubated for 2 h, spun, and washed 5 times with IP lysis buffer. Protein complexes were eluted by boiling with Laemmli buffer. For Figure 6C, immunoprecipitations were carried out as described above with α-Flag M2 (Sigma), then immunoblotted with α-SQ2 ([30]; kindly provided by S. Elledge), or α-ETV1 (Abcam).

### In Vitro Kinase Assays

To create GST-ETV1 (amino acids 1–290), the corresponding portion of ETV1 was PCR amplified using pEF6-Blast-3xFlag-ETV1 as a template and cloned into pGEX-4T-3 (GE Healthcare). The construct was confirmed by sequencing. For the smaller GST-ETV1 fusion proteins, synthetic oligos corresponding to amino acids 9–23 (SQ1), 44–58 (SQ2), 97–111 (SQ3), 165–179 (SQ4) or 240–254 (SQ5) were annealed and cloned into pGEX-4T-3. In vitro kinase assays were performed as previously described [62] except that reaction volumes were quadrupled. ^32^P-labeled products were visualized by autoradiography.

### Chromatin Immunoprecipitation Assays

ChIP assays were carried out as described previously [63], [64] with the following minor modifications. Briefly, 5×10^7^ cells were first incubated with ethylene glycol bis(succinimidyl succinate) (EGS) for 30 min and then incubated with 1% formaldehyde for 10 min at room temperature before crosslinking was quenched by addition of 0.125 M glycine. Cells were collected by centrifugation and lysed in lysis buffer containing 50 mM Tris–HCl pH 8.0, 10 mM EDTA, 0.5% SDS, proteinase inhibitors (Roche) and phosphatase inhibitors (Sigma). The cell suspension was sonicated for 15 min total time with 30 seconds ON and 30 seconds OFF using Bioruptor (Diagenode). Sonicated chromatin was then incubated at 4°C overnight with 5 µg of the appropriate antibody: α-ATR (Abcam), α-ETV1 (Abcam), α-E2F1 (Santa Cruz), α-MYC (Cell Signaling Technology), α-p53 (Santa Cruz), α-SP1 (Abcam), and corresponding IgG control. Immunoprecipitated chromatin DNA was analyzed by real-time PCR using the following primers: *TERT* promoter (−3 kb) (for 5′-ACGATGGAGGCAGTCAGTCT-3′; rev 5′-T CCCCACACACTTCATGCTA-3′), *TERT* promoter (−300 bp) (for 5′-GTTCCCAGGGCCTCCA CATC-3′; rev 5′-GCGGAGAGAGGTCGAATCGG-3′), *TERT* intron 1 (0.4 kb) (for 5′-GAACC AGCGACATGCGGAGAGCA-3′; rev: 5′-AGCTCCTTCAGGCAGGACACCT-3′). Fold enrichment was calculated by comparing the amplification threshold (Ct) value of a given ChIP sample with that obtained in the IgG control at the same target locus. All experiments were performed at least 2–3 times in either duplicate or triplicate.

### Immunofluorescence

Quantification of γ-H2AX-positive cells was performed as previously described [65] with modifications. Briefly, cells were seeded onto 22-mm glass coverslips and 48 h later, the coverslips were washed in PBS, incubated in cytoskeleton buffer (10 mM piperazine-*N*,N′-bis[2-ethanesulfonic acid] [PIPES] pH 6.8, 100 mM NaCl, 300 mM sucrose, 3 mM MgCl_2_, 1 mM EGTA, 0.5% Triton X-100] for 5 min on ice. After several washes with ice-cold PBS, the cells were fixed in 4% paraformaldehyde for 20 min and permeabilized in 0.5% Triton X-100 solution for 15 min at room temperature. Cells were blocked with 2% BSA in PBS, incubated with primary antibody anti-phosphoH2AX (Ser139) (Millipore, JBW301) overnight at 4°C, washed three times with 1× PBS, and incubated with secondary antibody Cy3-conjugated sheep anti-mouse IgG (Sigma-Aldrich) for 1 h at room temperature. Cells were then washed, counterstained with 4′,6′-diamidino-2-phenylindole (DAPI), and mounted in 90% glycerol and 2% 1,4-diaza-bicyclo-(2,2,2)-octane (DABCO). Images were captured using a Zeiss AxioCam HRc camera, and 10 fields of cells were counted for each sample in duplicate.

## Supporting Information

Figure S1 Characterization of p53 function in the human cancer cell lines used in this study. Immunoblot analysis monitoring p21 levels in cell lines treated with 5-fluorouracil (5-FU) or etoposide. The results show that in all p53+ cells, p21 levels increased following treatment with either DNA damaging agent, indicative of functional p53. By contrast, in all p53− cells p21 levels were reduced or undetectable following treatment with either DNA damaging agent, confirming the absence of functional p53.
(TIF)

Figure S2 Analysis of target gene expression following shRNA- or siRNA-mediated knockdown in p53− HCT116 cells. (A) p53− HCT116 cells were infected with a lentivirus expressing each individual candidate shRNA, or as a control a non-silencing (NS) shRNA, and target gene expression was analyzed by quantitative RT-PCR (qRT-PCR). Target gene expression was normalized to that obtained with the NS shRNA, which was set to 1. Error bars represent SD. (B) qRT-PCR analysis of target gene expression in p53− HCT116 cells transfected with an siRNA directed against an individual candidate gene or a control lamin A/C (LMNA) siRNA. Target gene expression was normalized to that obtained with the LMNA siRNA, which was set to 1. Error bars represent SD.
(TIF)

Figure S3 Comparison of single versus multiple siRNA knockdowns on proliferation and TERT expression in p53− cell lines. (A) (Left) Proliferation of p53− HCT116 cells transfected with single or multiple siRNAs as indicated was determined by an Alamar Blue fluorescence assay. The results were normalized to that obtained with the lamin A/C (LMNA) siRNA, which was set to 1. Error bars represent SD. (Right) Immunoblot analysis monitoring TERT levels in p53− HCT116 cells transfected with single or multiple siRNAs as indicated. α-tubulin (TUBA) was monitored as a loading control. (B) Proliferation of p53− A549 cells as described in panel A (left). (C) Proliferation of p53− RKO cells as described in panel A (left). The results show that the effects of double knockdowns were very similar to those observed following single knockdowns.
(TIF)

Figure S4 Confirmation of inhibition of ATR by CGK733 and ETP46464. Immunoblot analysis monitoring phospho-CHK1 (Ser317) levels in UV-irradiated (50 J/m^2^) p53+ and p53− HCT116 cells treated with CGK733 (top; 0, 2, 3, 4, 5 and 6 µM) or ETP46464 (bottom; 0, 0.5, 1, 2, 4 and 8 µM). α-tubulin (TUBA) and β-actin (ACTB) were monitored as loading controls. The results show that the levels of phospho-CHK1 (Ser317), a target of ATR, were reduced following addition of either CGK733 or ETP46464, indicating ATR activity was inhibited.
(TIF)

Figure S5 Effect of knockdown of ETV1, ATR or TERT on senescence induction. (A) Representative images of p53+ and p53− HCT116 cells expressing a non-silencing (NS) shRNA or one of two unrelated TERT shRNAs and stained for senescence-associated β-galactosidase. (B) Representative images of p53+ and p53− HCT116 cells expressing a NS, ATR or ETV1 shRNA and stained for senescence-associated β-galactosidase. (C) Senescence-associated β-galactosidase assay in p53+ and p53− HCT116 cells expressing a NS, ATR, ETV1 or TERT shRNA. Senescence-associated β-galactosidase activity was normalized to that obtained using a NS shRNA, which was set to 1. In this experiment, which is a replicate of that shown in Figure 4A, 4B, the effects of ATR, ETV1 and TERT knockdown were analyzed simultaneously.
(TIF)

Figure S6 FACS analysis of p53+ and p53− HCT116 cells following knockdown of TERT, ATR or ETV1. (A) FACS analysis of p53+ and p53− HCT116 cells expressing a non-silencing (NS) shRNA or one of two unrelated TERT shRNAs. (B) FACS analysis of p53+ and p53− HCT116 cells expressing a NS shRNA or one of two unrelated ATR or ETV1 shRNAs. (C) FACS analysis of p53+ and p53− HCT116 cells expressing a NS, TERT, ATR or ETV1 shRNA. In this experiment, which is a replicate of that shown in panels A and B, the effects of ATR, ETV1 and TERT knockdown were analyzed simultaneously. The percentage of cells in G1, S and G2/M is indicated.
(TIF)

Figure S7 Restoration of proliferation following ectopic expression of TERT following ETV1 or ATR knockdown. (A) Proliferation of p53+ and p53− HCT116 cells transfected with a control (lamin A/C; LMNA), ATR or ETV1 siRNA and stably expressing TERT, or as a control GFP, was determined by counting cells. Cell number was normalized to that obtained using a LMNA siRNA in p53+/GFP cells, which was set to 1. Error bars represent SD. (B) Immunoblot analysis monitoring TERT levels in p53+ and p53− HCT116 cells stably transfected with a plasmid expressing TERT or, as a control, green fluorescent protein (GFP). The upper and lower bands represent ectopic FLAG-HA-TERT and endogenous TERT, respectively, as indicated. α-tubulin (TUBA) was monitored as a loading control.
(TIF)

Figure S8 In the absence of ATR kinase activity DNA damage does not stabilize ETV1. Immunoblot analysis monitoring ETV1 levels in p53− HCT116 cells in the presence or absence of irradiation with ultraviolet (UV) light or treatment with ETP46464 as indicated. α-tubulin (TUBA) was monitoring as a loading control.
(TIF)

Figure S9 ATR and ETV1 promote TERT expression in human cancer cell lines expressing a p53 mutant and in HeLa cells. (A) Immunoblot analysis monitoring TERT levels in various human cancer cell lines expressing a p53 mutant transfected with a non-silencing (NS), ATR or ETV1 shRNA. α-tubulin (TUBA) was monitoring as a loading control. (B) Immunoblot analysis monitoring TERT levels in HeLa cells transfected with a control (lamin A/C; LMNA), ATR or ETV1 siRNA. β-actin (ACTB) was monitored as a loading control. The results show that ATR and ETV1 are required for TERT expression human cancer cell lines expressing a p53 mutant (panel A) or in which wild type p53 is inactivated by expression of human papilloma virus E6 protein (panel B).
(TIF)

Figure S10 Confirmation of increased ETV1 levels upon ectopic expression. Immunoblot analysis monitoring FLAG-ETV1 levels in p53+ and p53− HCT116 cells stably transfected with a plasmid expressing FLAG-ETV1 or, as a control, empty vector. The upper band represents FLAG-ETV1, and the lower signal is a non-specific band.
(TIF)

Figure S11 Inhibition of ATR kinase activity results in loss of ETV1 SQ phosphorylation. p53+ HCT116 cells expressing FLAG-ETV1 were treated in the presence or absence of CGK733. Subsequently, extracts were prepared and immunoprecipitated using a FLAG antibody and the immunoprecipitate analyzed by immunoblotting using an antibody that recognizes ETV1 or a phosphorylated serine-glutamine motif (P-SQ).
(TIF)

Figure S12 Analysis of ETV1 occupancy on the *TERT* promoter following treatment with ETP46464 or UV irradation. (A) ChIP analysis monitoring ETV1 occupancy at two regions of the *TERT* promoter, in the first intron or 3 kb upstream of the transcription start-site, in p53+ and p53− HCT116 cells treated in the presence or absence of ETP46464 (5 µM). Error bars represent SD. The results confirm the ATR dependency of ETV1 binding to the *TERT* promoter, shown in Figure 7A, using a different ATR inhibitor. (B) ChIP analysis monitoring ETV1 occupancy in the *TERT* first intron in p53− HCT116 cells that were or were not irradiated with UV light (50 J/m^2^) in the presence or absence of ETP46464 (5 µM). The results show that DNA damage, which activates ATR, modestly increases binding of ETV1 to the *TERT* promoter.
(TIF)

Figure S13 ETV1 can be ectopically expressed at detectable levels in ATR knockdown cells. Immunoblot analysis monitoring FLAG-ETV1 levels in p53− HCT116 cells stably transfected with a plasmid expressing FLAG-ETV1 or, as a control, empty vector and also transfected with a control (lamin A/C; LMNA) or one of two independent ATR siRNAs as indicated. β-actin (ACTB) was monitored as a loading control.
(TIF)

Figure S14 Ectopic expression of ETV1 requires TERT to restore proliferation in p53− HCT116 cells following ATR knockdown and does not induce DNA damage. (A) Proliferation of p53− HCT116 cells stably expressing ETV1, or the empty expression vector, were transfected with single or multiple siRNAs as indicated. Proliferation was determined by an Alamar Blue fluorescence assay. The results were normalized to that obtained with the control (lamin A/C; LMNA) siRNA, which was set to 1. Error bars represent SD. (B) p53− HCT116 cells stably expressing ETV1, or the empty expression vector, were transfected with a non-silencing (NS) or ATR shRNA. Cells were stained for γ-H2AX, a marker of double-strand breaks (DNA damage), and analyzed by fluorescence microscopy. Error bars represent SD.
(TIF)

Figure S15 Analysis of E2F1, MYC, SP1 and p53 occupancy on the *TERT* promoter in p53+ and p53− HCT116 cells. (A–D) ChIP analysis in p53+ and p53− HCT116 cells monitoring occupancy of E2F1 (A), MYC (B), SP1 (C) and p53 (D) at three regions of the *TERT* promoter: in the first intron, or 300 bp or 3 kb upstream of the transcription start-site. Error bars represent SD.
(TIF)

Figure S16 ATR kinase activity is not required for TERT expression in human MCF10A cells expressing a dominant-negative p53 mutant or in p53− mouse embryo fibroblasts. (A) (Left) Immunoblot analysis monitoring TERT and ETV1 levels in human MCF10A cells stably expressing a p53 dominant-negative mutant (p53-DD), or the empty expression vector, treated in the presence or absence of ETP46464. β-actin (ACTB) was monitored as a loading control. (Right) Immunoblot analysis monitoring the level of the p53 dominant-negative mutant in the MCF10A stable cell lines used in panel A. (B) Immunoblot analysis monitoring TERT and ETV1 levels in p53+ and p53− mouse embryo fibroblasts (MEFs) treated in the presence or absence of ETP46464. α-tubulin (TUBA) was monitored as a loading control.
(TIF)

Table S1 List of 103 genes identified in the genome-wide RNAi screen for genes preferentially required for proliferation of p53− human cancer cell lines.
(DOC)

Table S2 Summary of the cell culture results in Figure 1 and Figure 2.
(DOC)

Table S3 Basis for the p53− status in each of the p53− cell lines used in this study.
(DOC)

Table S4 Oligo ID numbers and locations for shRNAs obtained from Open Biosystems, sequences of synthesized siRNAs, and primer sequences for qRT-PCR analysis.
(DOC)
