# Supplementary figures and images for: A Synthetic Interaction Screen Identifies Factors Selectively Required for Proliferation and TERT Transcription in p53-Deficient Human Cancer Cells
Source: PLoS Genet. 2012 Dec 20;8(12):e1003151. doi: 10.1371/journal.pgen.1003151 (PMC3527276; doi:10.1371/journal.pgen.1003151)

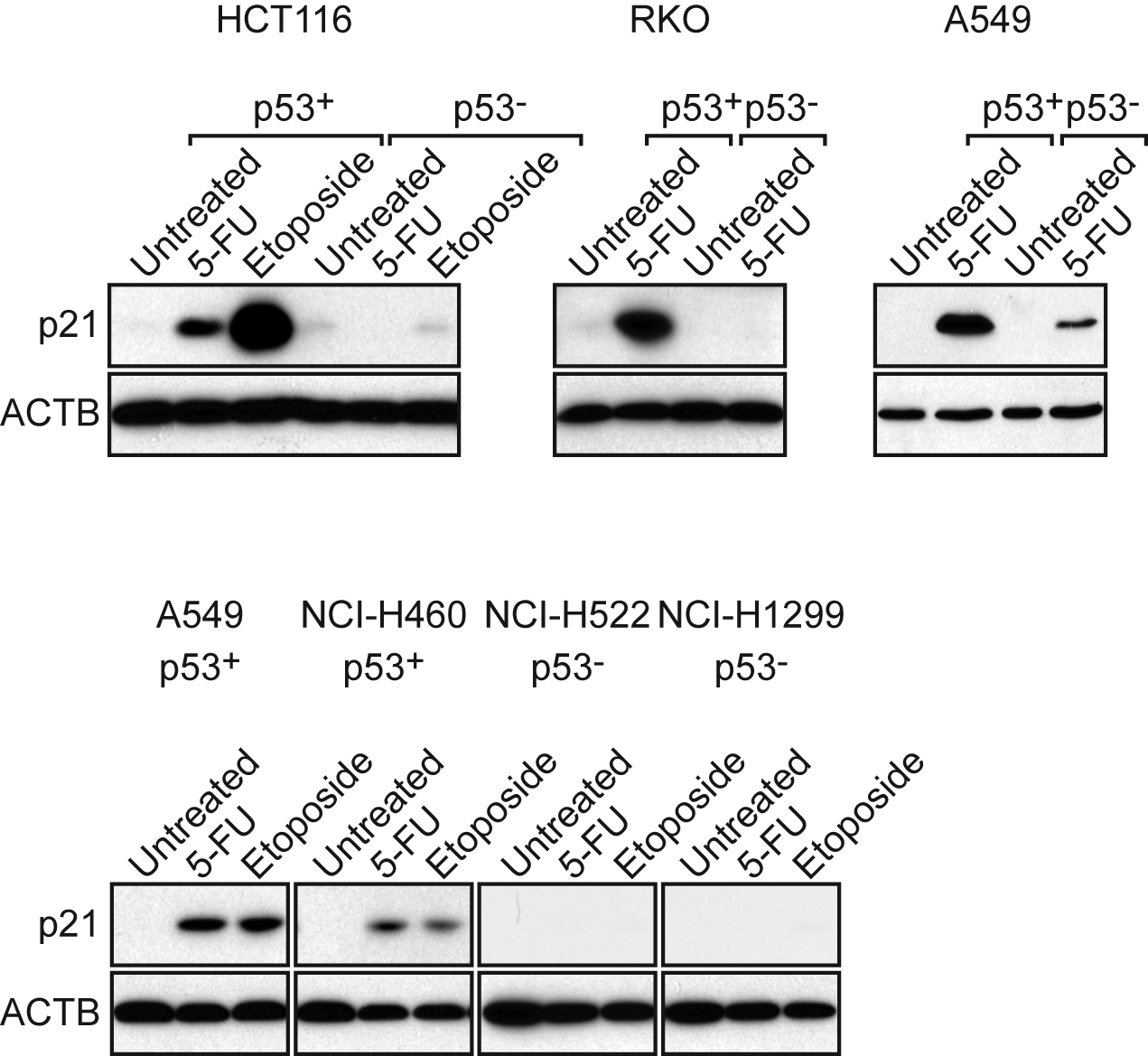

Supplement: Figure S1 — Characterization of p53 function in the human cancer cell lines used in this study. Immunoblot analysis monitoring p21 levels in cell lines treated with 5-fluorouracil (5-FU) or etoposide. The results show that in all p53+ cells, p21 levels increased following treatment with either DNA damaging agent, indicative of functional p53. By contrast, in all p53− cells p21 levels were reduced or undetectable following treatment with either DNA damaging agent, confirming the absence of functional p53. (TIF) [file pgen.1003151.s001.tif]

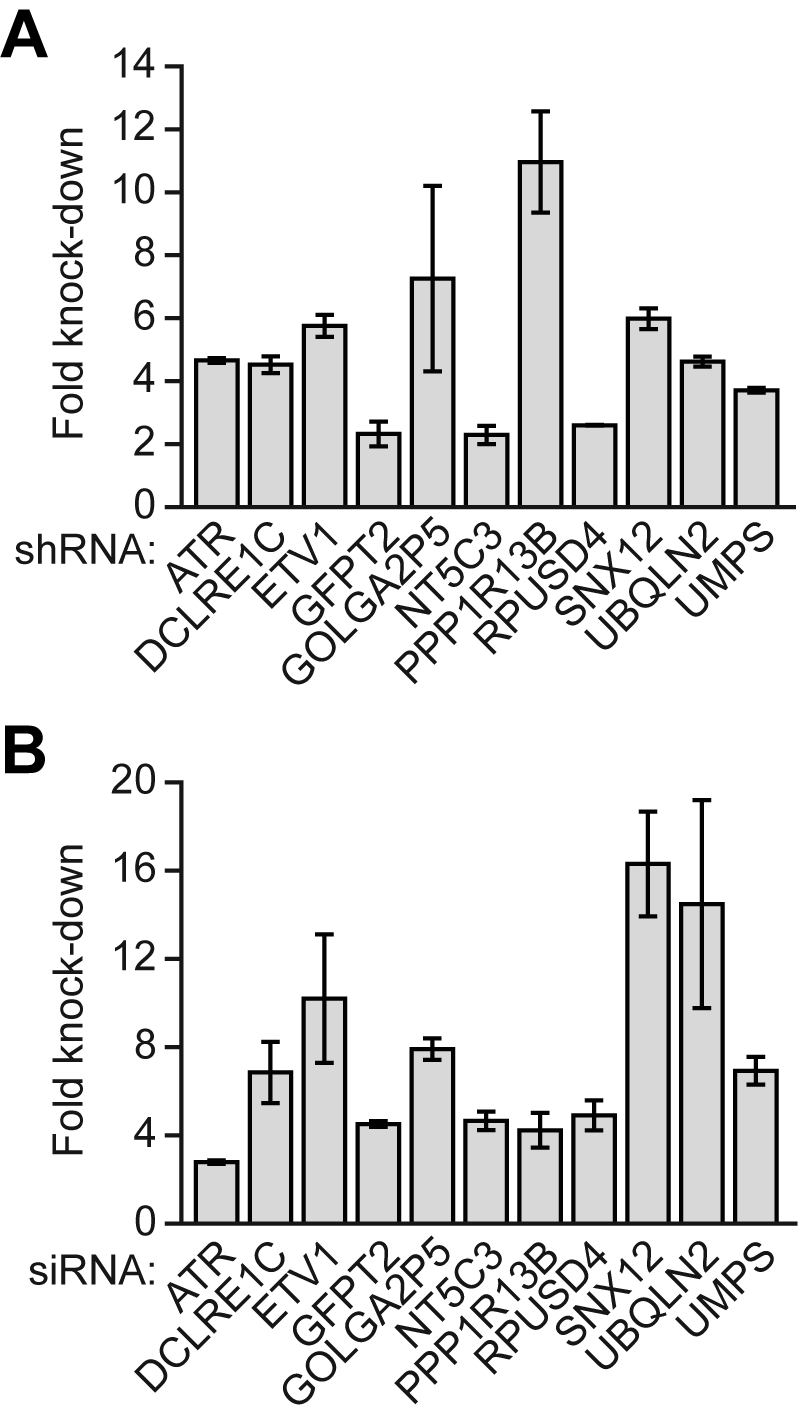

Supplement: Figure S2 — Analysis of target gene expression following shRNA- or siRNA-mediated knockdown in p53− HCT116 cells. (A) p53− HCT116 cells were infected with a lentivirus expressing each individual candidate shRNA, or as a control a non-silencing (NS) shRNA, and target gene expression was analyzed by quantitative RT-PCR (qRT-PCR). Target gene expression was normalized to that obtained with the NS shRNA, which was set to 1. Error bars represent SD. (B) qRT-PCR analysis of target gene expression in p53− HCT116 cells transfected with an siRNA directed against an individual candidate gene or a control lamin A/C (LMNA) siRNA. Target gene expression was normalized to that obtained with the LMNA siRNA, which was set to 1. Error bars represent SD. (TIF) [file pgen.1003151.s002.tif]

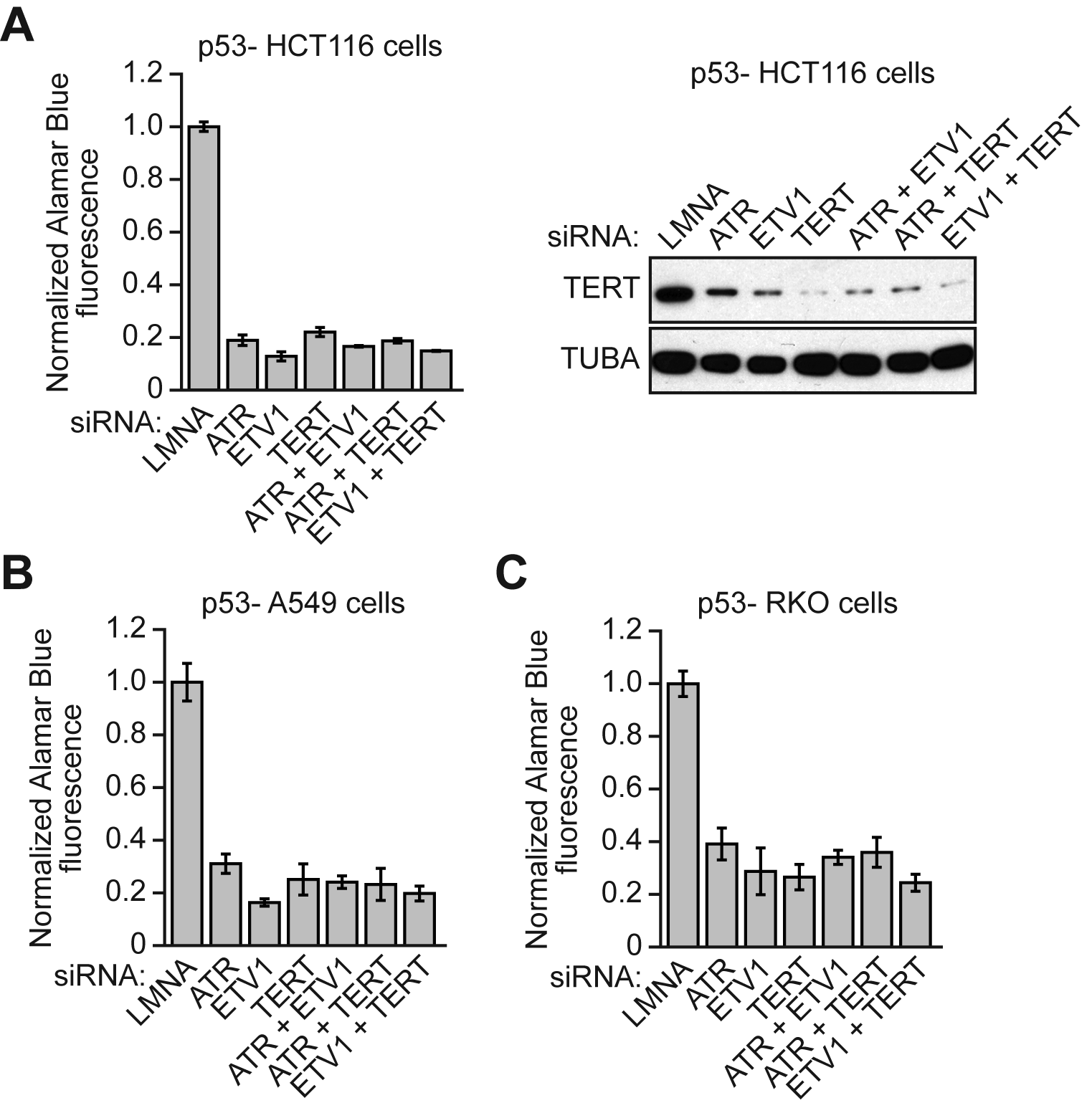

Supplement: Figure S3 — Comparison of single versus multiple siRNA knockdowns on proliferation and TERT expression in p53− cell lines. (A) (Left) Proliferation of p53− HCT116 cells transfected with single or multiple siRNAs as indicated was determined by an Alamar Blue fluorescence assay. The results were normalized to that obtained with the lamin A/C (LMNA) siRNA, which was set to 1. Error bars represent SD. (Right) Immunoblot analysis monitoring TERT levels in p53− HCT116 cells transfected with single or multiple siRNAs as indicated. α-tubulin (TUBA) was monitored as a loading control. (B) Proliferation of p53− A549 cells as described in panel A (left). (C) Proliferation of p53− RKO cells as described in panel A (left). The results show that the effects of double knockdowns were very similar to those observed following single knockdowns. (TIF) [file pgen.1003151.s003.tif]

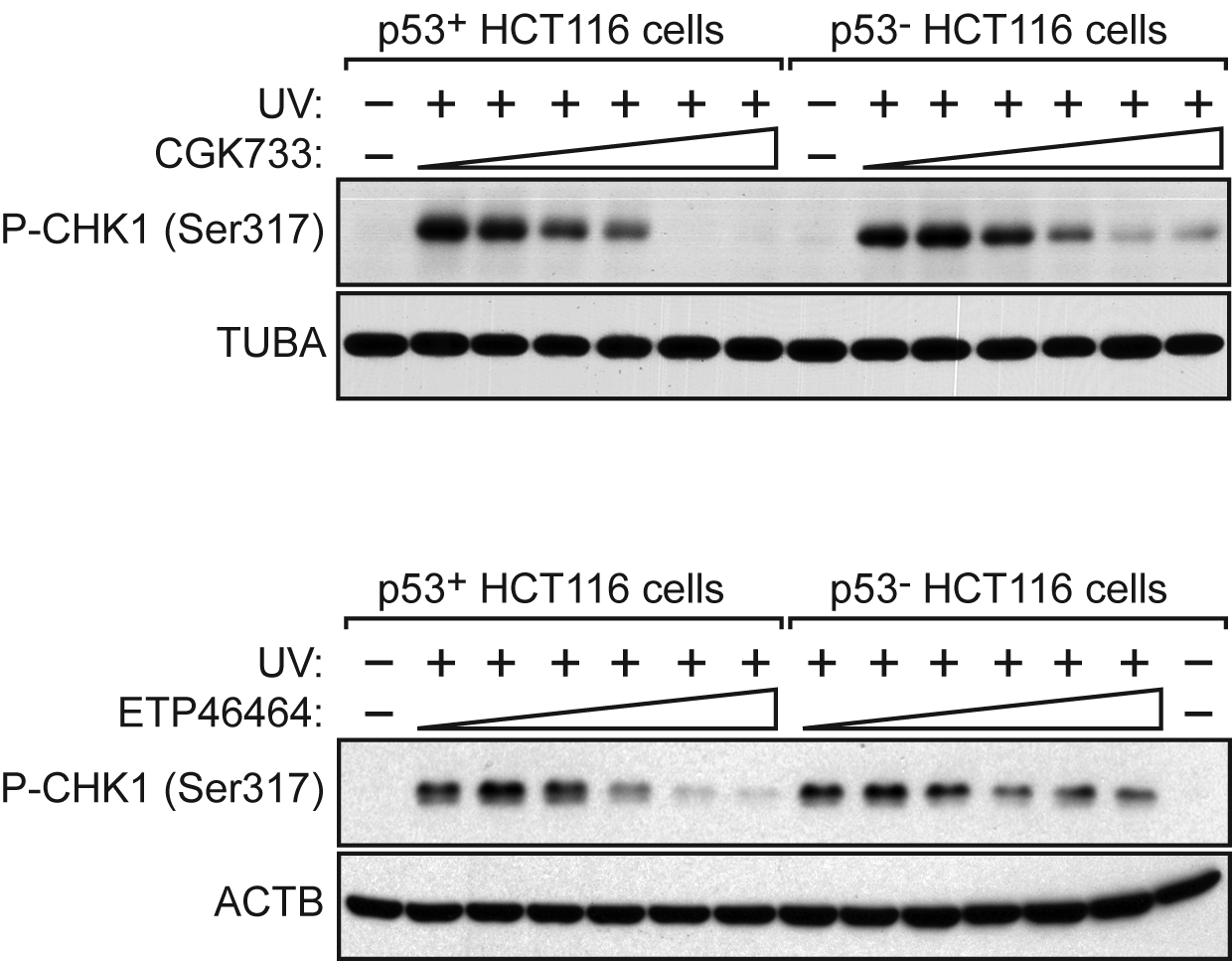

Supplement: Figure S4 — Confirmation of inhibition of ATR by CGK733 and ETP46464. Immunoblot analysis monitoring phospho-CHK1 (Ser317) levels in UV-irradiated (50 J/m2) p53+ and p53− HCT116 cells treated with CGK733 (top; 0, 2, 3, 4, 5 and 6 µM) or ETP46464 (bottom; 0, 0.5, 1, 2, 4 and 8 µM). α-tubulin (TUBA) and β-actin (ACTB) were monitored as loading controls. The results show that the levels of phospho-CHK1 (Ser317), a target of ATR, were reduced following addition of either CGK733 or ETP46464, indicating ATR activity was inhibited. (TIF) [file pgen.1003151.s004.tif]

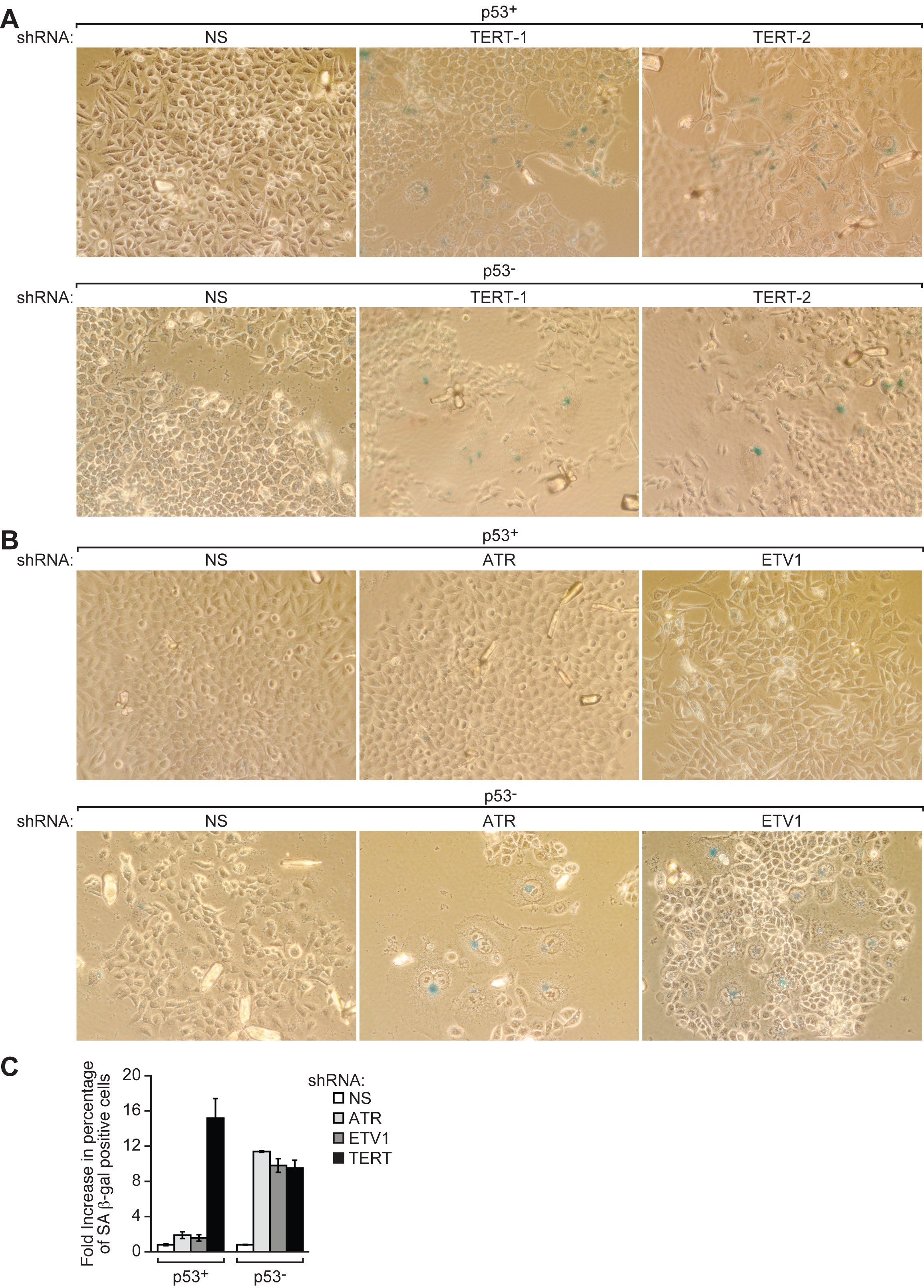

Supplement: Figure S5 — Effect of knockdown of ETV1, ATR or TERT on senescence induction. (A) Representative images of p53+ and p53− HCT116 cells expressing a non-silencing (NS) shRNA or one of two unrelated TERT shRNAs and stained for senescence-associated β-galactosidase. (B) Representative images of p53+ and p53− HCT116 cells expressing a NS, ATR or ETV1 shRNA and stained for senescence-associated β-galactosidase. (C) Senescence-associated β-galactosidase assay in p53+ and p53− HCT116 cells expressing a NS, ATR, ETV1 or TERT shRNA. Senescence-associated β-galactosidase activity was normalized to that obtained using a NS shRNA, which was set to 1. In this experiment, which is a replicate of that shown in Figure 4A, 4B, the effects of ATR, ETV1 and TERT knockdown were analyzed simultaneously. (TIF) [file pgen.1003151.s005.tif]

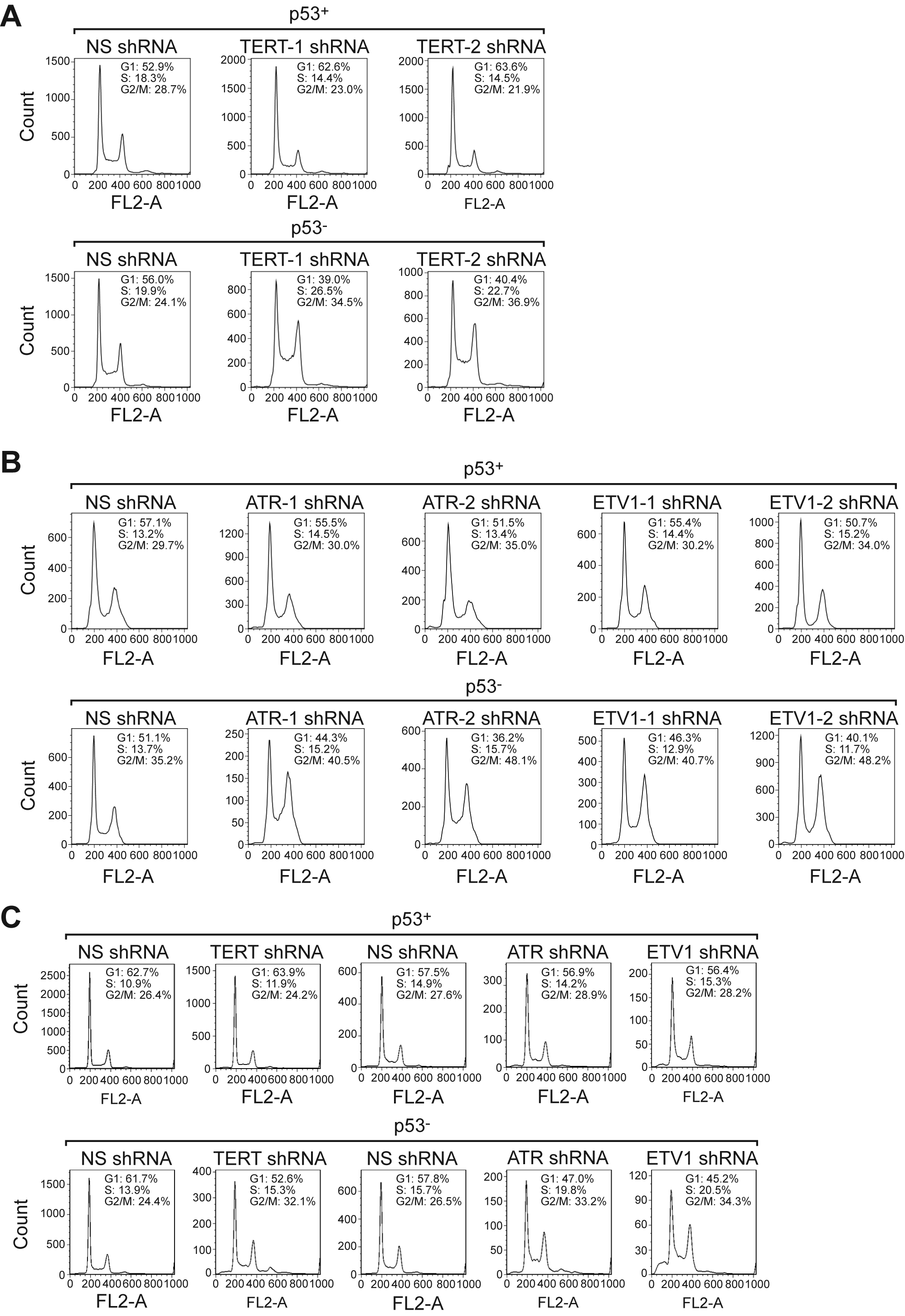

Supplement: Figure S6 — FACS analysis of p53+ and p53− HCT116 cells following knockdown of TERT, ATR or ETV1. (A) FACS analysis of p53+ and p53− HCT116 cells expressing a non-silencing (NS) shRNA or one of two unrelated TERT shRNAs. (B) FACS analysis of p53+ and p53− HCT116 cells expressing a NS shRNA or one of two unrelated ATR or ETV1 shRNAs. (C) FACS analysis of p53+ and p53− HCT116 cells expressing a NS, TERT, ATR or ETV1 shRNA. In this experiment, which is a replicate of that shown in panels A and B, the effects of ATR, ETV1 and TERT knockdown were analyzed simultaneously. The percentage of cells in G1, S and G2/M is indicated. (TIF) [file pgen.1003151.s006.tif]

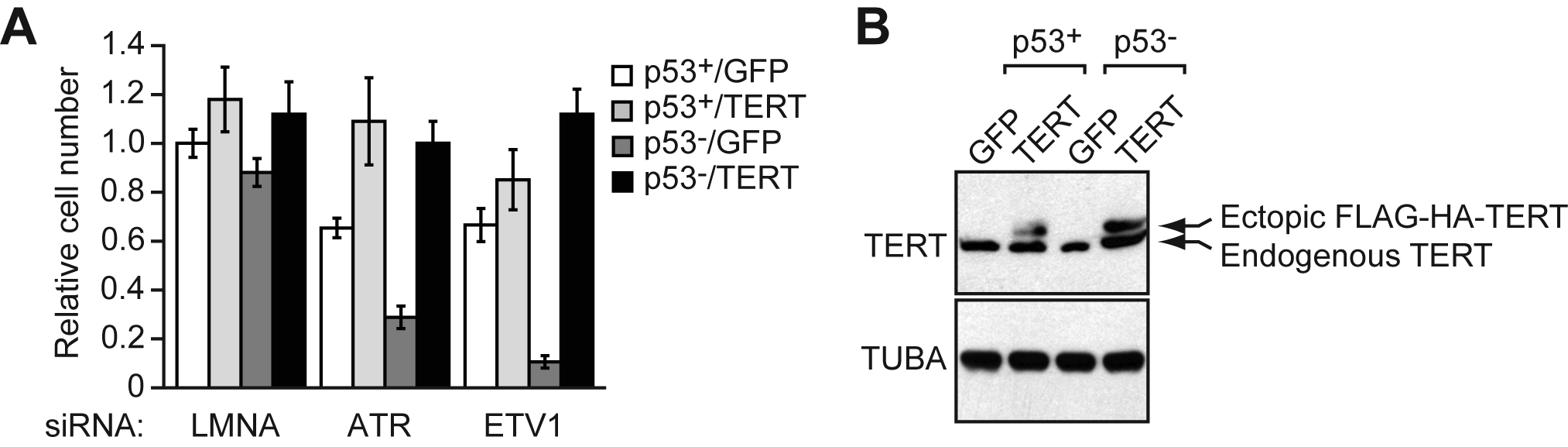

Supplement: Figure S7 — Restoration of proliferation following ectopic expression of TERT following ETV1 or ATR knockdown. (A) Proliferation of p53+ and p53− HCT116 cells transfected with a control (lamin A/C; LMNA), ATR or ETV1 siRNA and stably expressing TERT, or as a control GFP, was determined by counting cells. Cell number was normalized to that obtained using a LMNA siRNA in p53+/GFP cells, which was set to 1. Error bars represent SD. (B) Immunoblot analysis monitoring TERT levels in p53+ and p53− HCT116 cells stably transfected with a plasmid expressing TERT or, as a control, green fluorescent protein (GFP). The upper and lower bands represent ectopic FLAG-HA-TERT and endogenous TERT, respectively, as indicated. α-tubulin (TUBA) was monitored as a loading control. (TIF) [file pgen.1003151.s007.tif]

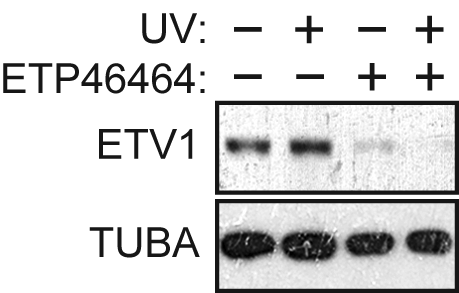

Supplement: Figure S8 — In the absence of ATR kinase activity DNA damage does not stabilize ETV1. Immunoblot analysis monitoring ETV1 levels in p53− HCT116 cells in the presence or absence of irradiation with ultraviolet (UV) light or treatment with ETP46464 as indicated. α-tubulin (TUBA) was monitoring as a loading control. (TIF) [file pgen.1003151.s008.tif]

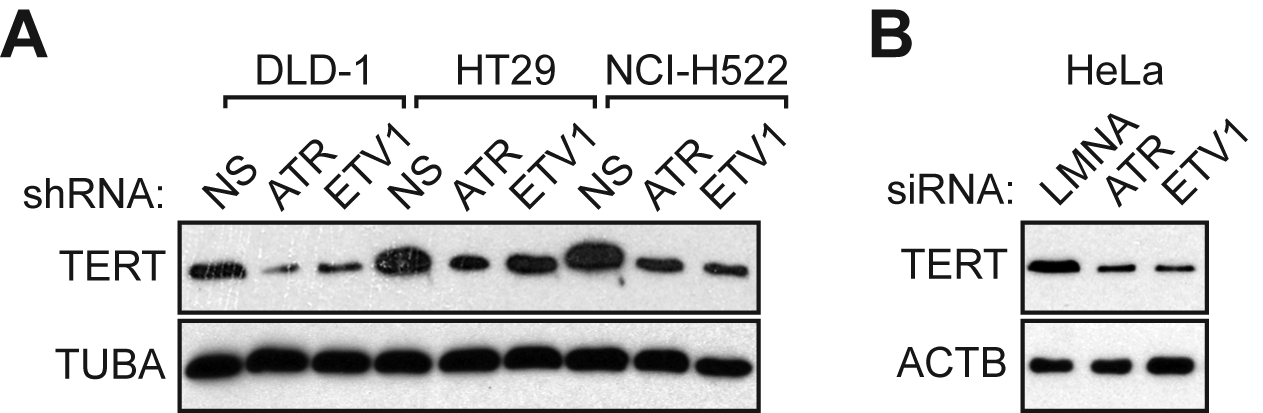

Supplement: Figure S9 — ATR and ETV1 promote TERT expression in human cancer cell lines expressing a p53 mutant and in HeLa cells. (A) Immunoblot analysis monitoring TERT levels in various human cancer cell lines expressing a p53 mutant transfected with a non-silencing (NS), ATR or ETV1 shRNA. α-tubulin (TUBA) was monitoring as a loading control. (B) Immunoblot analysis monitoring TERT levels in HeLa cells transfected with a control (lamin A/C; LMNA), ATR or ETV1 siRNA. β-actin (ACTB) was monitored as a loading control. The results show that ATR and ETV1 are required for TERT expression human cancer cell lines expressing a p53 mutant (panel A) or in which wild type p53 is inactivated by expression of human papilloma virus E6 protein (panel B). (TIF) [file pgen.1003151.s009.tif]

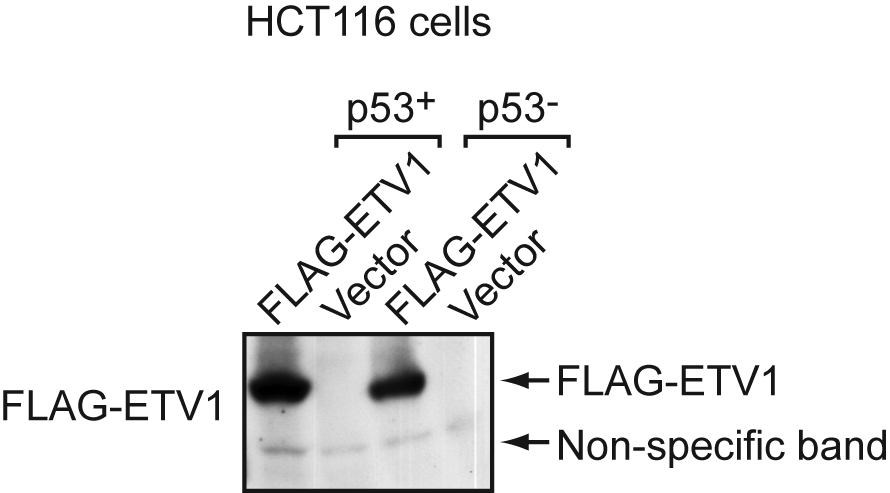

Supplement: Figure S10 — Confirmation of increased ETV1 levels upon ectopic expression. Immunoblot analysis monitoring FLAG-ETV1 levels in p53+ and p53− HCT116 cells stably transfected with a plasmid expressing FLAG-ETV1 or, as a control, empty vector. The upper band represents FLAG-ETV1, and the lower signal is a non-specific band. (TIF) [file pgen.1003151.s010.tif]

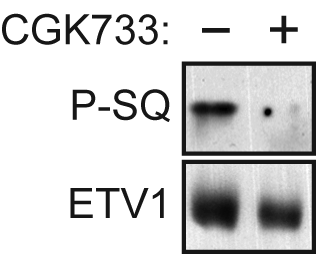

Supplement: Figure S11 — Inhibition of ATR kinase activity results in loss of ETV1 SQ phosphorylation. p53+ HCT116 cells expressing FLAG-ETV1 were treated in the presence or absence of CGK733. Subsequently, extracts were prepared and immunoprecipitated using a FLAG antibody and the immunoprecipitate analyzed by immunoblotting using an antibody that recognizes ETV1 or a phosphorylated serine-glutamine motif (P-SQ). (TIF) [file pgen.1003151.s011.tif]

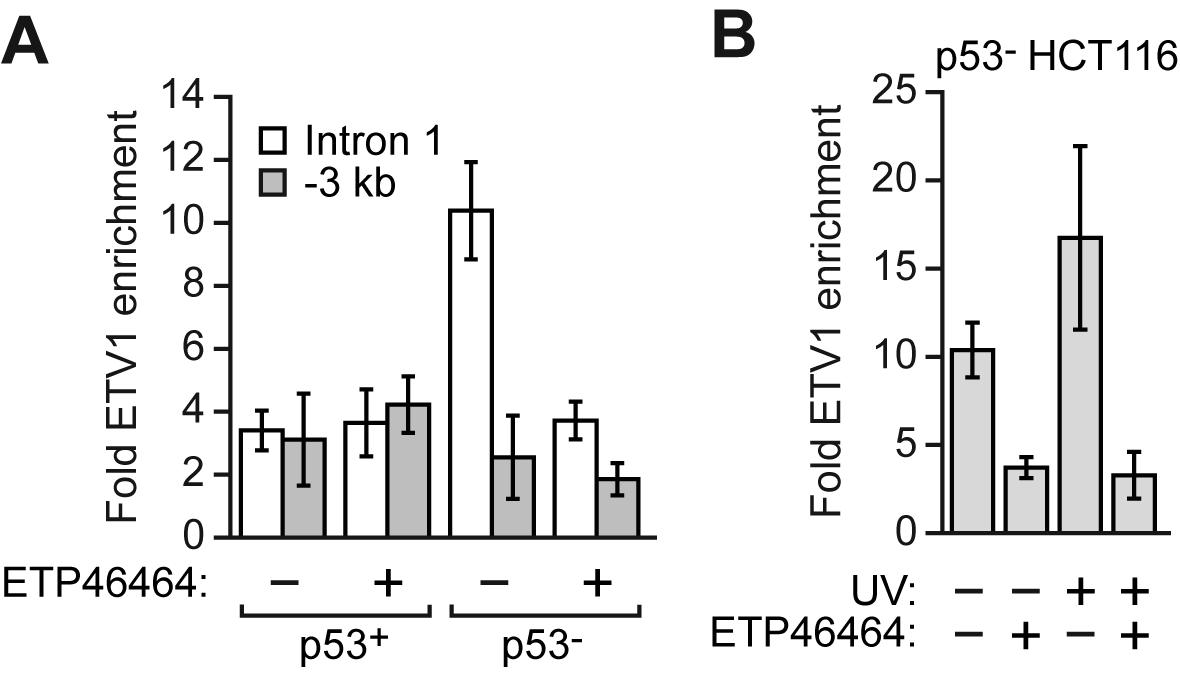

Supplement: Figure S12 — Analysis of ETV1 occupancy on the TERT promoter following treatment with ETP46464 or UV irradation. (A) ChIP analysis monitoring ETV1 occupancy at two regions of the TERT promoter, in the first intron or 3 kb upstream of the transcription start-site, in p53+ and p53− HCT116 cells treated in the presence or absence of ETP46464 (5 µM). Error bars represent SD. The results confirm the ATR dependency of ETV1 binding to the TERT promoter, shown in Figure 7A, using a different ATR inhibitor. (B) ChIP analysis monitoring ETV1 occupancy in the TERT first intron in p53− HCT116 cells that were or were not irradiated with UV light (50 J/m2) in the presence or absence of ETP46464 (5 µM). The results show that DNA damage, which activates ATR, modestly increases binding of ETV1 to the TERT promoter. (TIF) [file pgen.1003151.s012.tif]

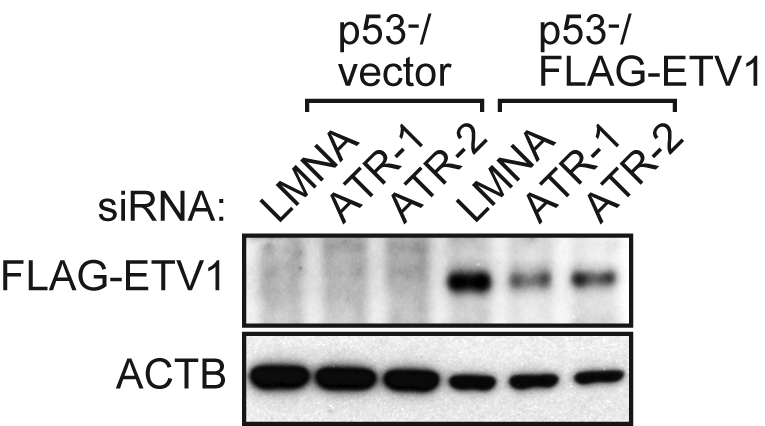

Supplement: Figure S13 — ETV1 can be ectopically expressed at detectable levels in ATR knockdown cells. Immunoblot analysis monitoring FLAG-ETV1 levels in p53− HCT116 cells stably transfected with a plasmid expressing FLAG-ETV1 or, as a control, empty vector and also transfected with a control (lamin A/C; LMNA) or one of two independent ATR siRNAs as indicated. β-actin (ACTB) was monitored as a loading control. (TIF) [file pgen.1003151.s013.tif]

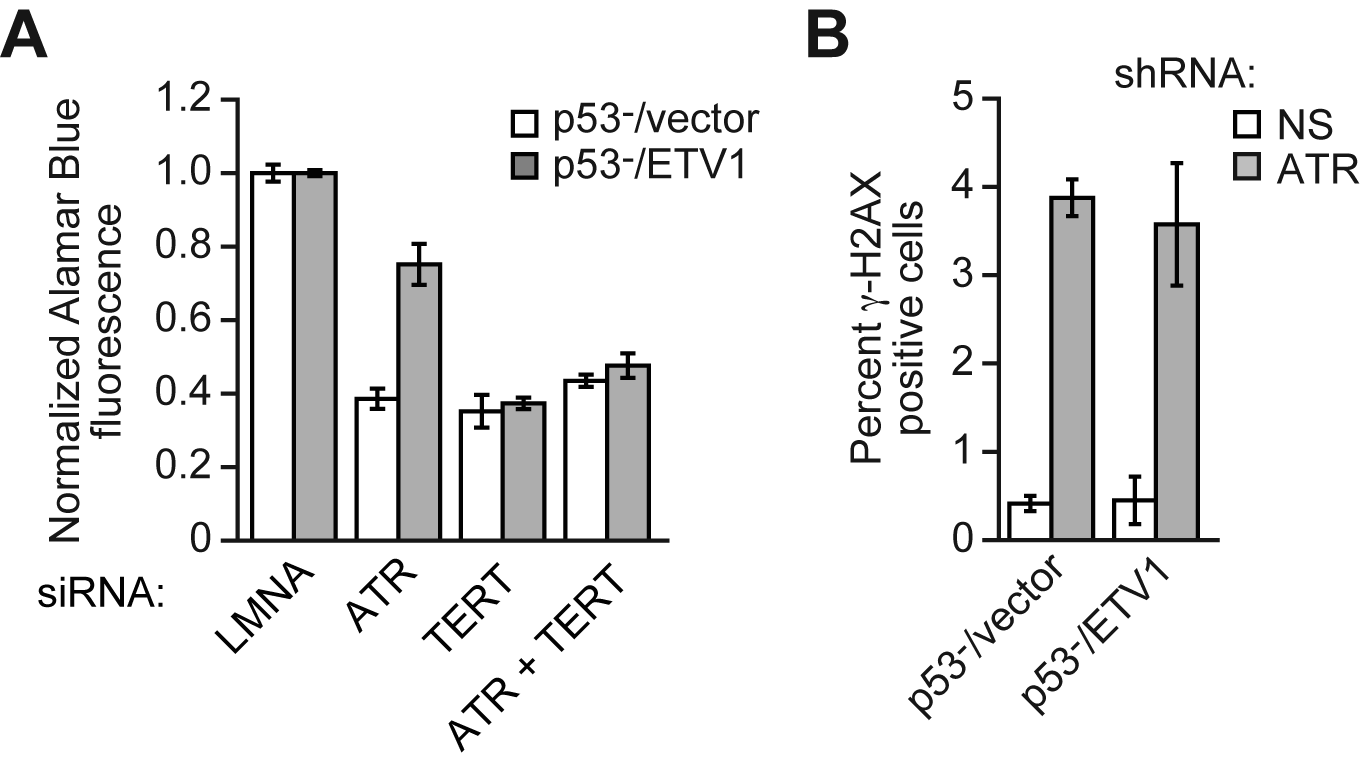

Supplement: Figure S14 — Ectopic expression of ETV1 requires TERT to restore proliferation in p53− HCT116 cells following ATR knockdown and does not induce DNA damage. (A) Proliferation of p53− HCT116 cells stably expressing ETV1, or the empty expression vector, were transfected with single or multiple siRNAs as indicated. Proliferation was determined by an Alamar Blue fluorescence assay. The results were normalized to that obtained with the control (lamin A/C; LMNA) siRNA, which was set to 1. Error bars represent SD. (B) p53− HCT116 cells stably expressing ETV1, or the empty expression vector, were transfected with a non-silencing (NS) or ATR shRNA. Cells were stained for γ-H2AX, a marker of double-strand breaks (DNA damage), and analyzed by fluorescence microscopy. Error bars represent SD. (TIF) [file pgen.1003151.s014.tif]

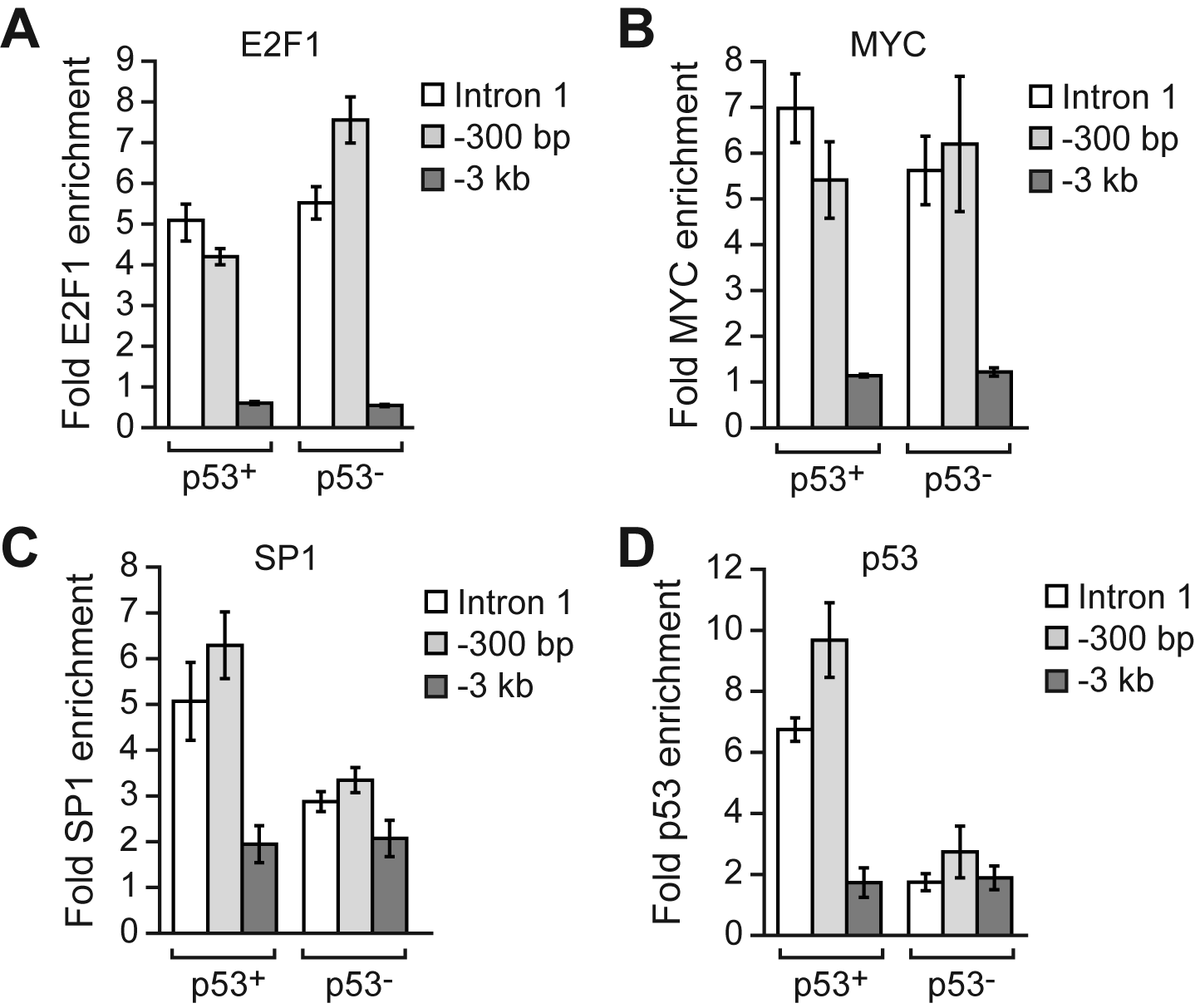

Supplement: Figure S15 — Analysis of E2F1, MYC, SP1 and p53 occupancy on the TERT promoter in p53+ and p53− HCT116 cells. (A–D) ChIP analysis in p53+ and p53− HCT116 cells monitoring occupancy of E2F1 (A), MYC (B), SP1 (C) and p53 (D) at three regions of the TERT promoter: in the first intron, or 300 bp or 3 kb upstream of the transcription start-site. Error bars represent SD. (TIF) [file pgen.1003151.s015.tif]

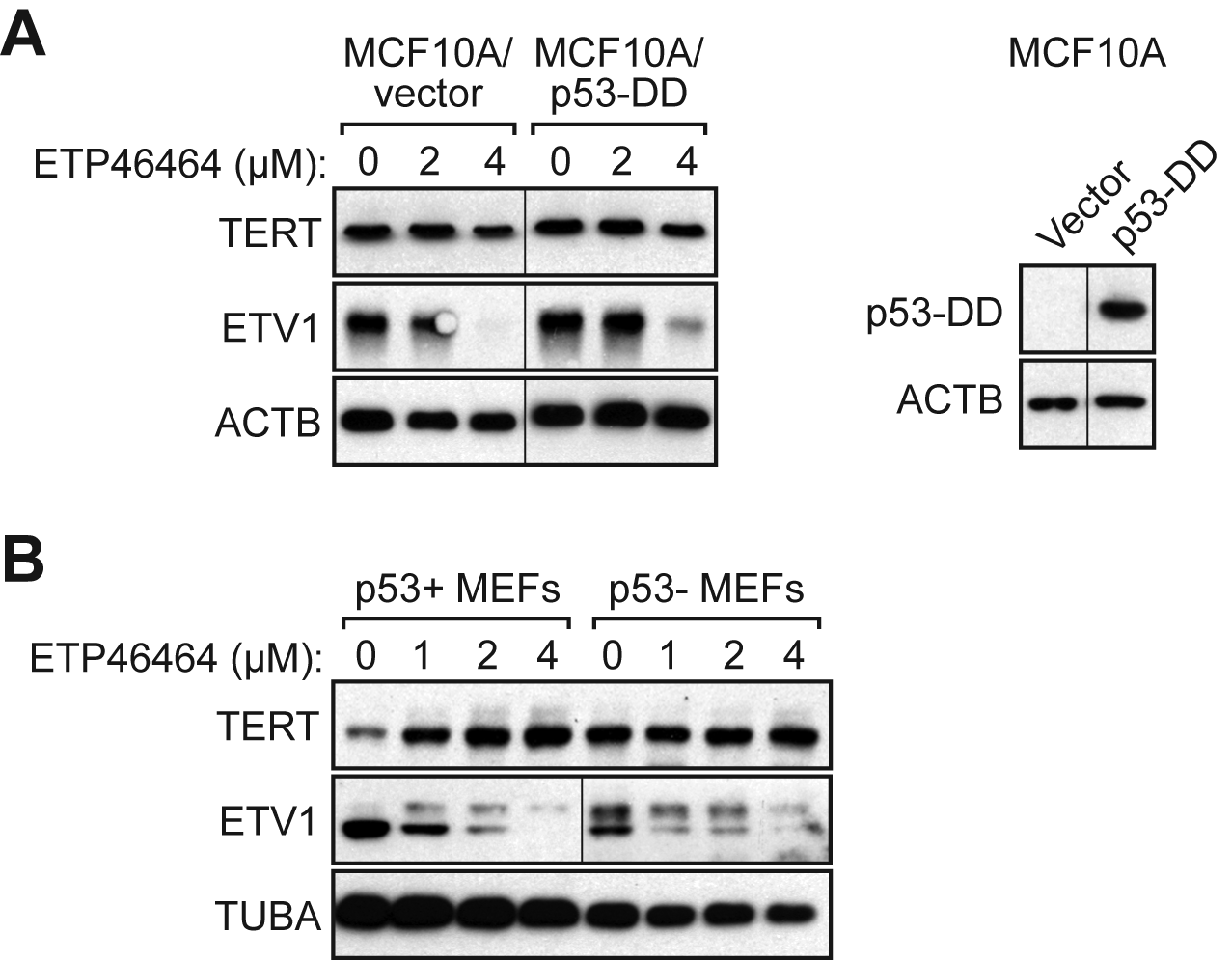

Supplement: Figure S16 — ATR kinase activity is not required for TERT expression in human MCF10A cells expressing a dominant-negative p53 mutant or in p53− mouse embryo fibroblasts. (A) (Left) Immunoblot analysis monitoring TERT and ETV1 levels in human MCF10A cells stably expressing a p53 dominant-negative mutant (p53-DD), or the empty expression vector, treated in the presence or absence of ETP46464. β-actin (ACTB) was monitored as a loading control. (Right) Immunoblot analysis monitoring the level of the p53 dominant-negative mutant in the MCF10A stable cell lines used in panel A. (B) Immunoblot analysis monitoring TERT and ETV1 levels in p53+ and p53− mouse embryo fibroblasts (MEFs) treated in the presence or absence of ETP46464. α-tubulin (TUBA) was monitored as a loading control. (TIF) [file pgen.1003151.s016.tif]
