# Supplementary material for: A Synthetic Interaction Screen Identifies Factors Selectively Required for Proliferation and TERT Transcription in p53-Deficient Human Cancer Cells
Source: PLoS Genet. 2012 Dec 20;8(12):e1003151. doi: 10.1371/journal.pgen.1003151 (PMC3527276; doi:10.1371/journal.pgen.1003151)
Supplement: Table S1 — List of 103 genes identified in the genome-wide RNAi screen for genes preferentially required for proliferation of p53− human cancer cell lines. (DOC) [file pgen.1003151.s017.doc]

**Table S1** List of 103 genes identified in the genome-wide RNAi screen for genes preferentially required for proliferation of p53- human cancer cell lines.

| **Gene symbol** | **Gene name** |
| --- | --- |
| *AAGAB* | alpha- and gamma-adaptin-binding protein |
| *ACVR1C* | activin A receptor, type 1C |
| *ADAM28* | ADAM metallopeptidase domain 28 |
| *ARL3* | ADP-ribosylation factor-like 3 |
| *ARMCX5* | armadillo repeat containing, X-linked 5 |
| *ARPP21* | cAMP-regulated phosphoprotein, 21 kDa |
| *ATR* | ataxia telangiectasia and Rad3 related |
| *CENPA* | centromere protein A |
| *CLCA3P* | chloride channel accessory 3, pseudogene |
| *CPA5* | carboxypeptidase A5 |
| *CYP20A1* | cytochrome P450, family 20, subfamily A, polypeptide 1 |
| *C12orf49* | chromosome 12 open reading frame 49 |
| *DCLRE1C* | DNA cross-link repair 1C |
| *DDX50* | DEAD (Asp-Glu-Ala-Asp) box polypeptide 50 |
| *DKK2* | dickkopf homolog 2 (Xenopus laevis) |
| *DOT1L* | DOT1-like, histone H3 methyltransferase (S. cerevisiae) |
| *DPP6* | dipeptidyl-peptidase 6 |
| *DZIP1* | DAZ-interacting protein 1 |
| *EGFR* | epidermal growth factor receptor |
| *ELL3* | elongation factor RNA polymerase II-like 3 |
| *EPG5* | ectopic P-granules autophagy protein 5 homolog (C. elegans) |
| *ETV1* | ets variant 1 |
| *EXOSC4* | exosome component 4 |
| *FAM82B* | family with sequence similarity 82, member B |
| *FHIT* | fragile histidine triad |
| *GABRG3* | gamma-aminobutyric acid (GABA) A receptor, gamma 3 |
| *GFPT2* | glutamine-fructose-6-phosphate transaminase 2 |
| *GK2* | glycerol kinase 2 |
| *GOLGA2P5* | golgin A2 pseudogene 5 |
| *GPSM3* | G-protein-signaling modulator 3 |
| *GRIN2B* | glutamate receptor, ionotropic, N-methyl D-aspartate 2B |
| *HAUS1* | HAUS augmin-like complex, subunit 1 |
| *HAUS4* | HAUS augmin-like complex, subunit 4 |
| *HINT3* | histidine triad nucleotide binding protein 3 |
| *HOXA9* | homeobox A9 |
| *HYAL4* | hyaluronoglucosaminidase 4 |
| *IL1A* | interleukin 1, alpha |
| *IL8* | interleukin 8 |
| *IMP3* | U3 small nucleolar ribonucleoprotein, homolog (yeast) |
| *INSIG1* | insulin-induced gene 1 |
| *IRX1* | iroquois homeobox 1 |
| *JMJD8* | jumonji domain containing 8 |
| *KBTBD5* | kelch repeat and BTB (POZ) domain containing 5 |
| *KLRF1* | killer cell lectin-like receptor subfamily F member 1 |
| *LAPTM5* | lysosomal protein transmembrane 5 |
| *LAP3* | leucine aminopeptidase 3 |
| *LGI4* | leucine-rich repeat LGI family, member 4 |
| *MCM8* | minichromosome maintenance complex component 8 |
| *MED8* | mediator complex subunit 8 |
| *MED22* | mediator complex subunit 22 |
| *MFSD12* | major facilitator superfamily domain containing 12 |
| *MIA2* | melanoma inhibitory activity 2 |
| *MORN2* | MORN repeat containing 2 |
| *MYBPC1* | myosin binding protein C, slow type |
| *MYOT* | myotilin |
| *NEUROG2* | neurogenin 2 |
| *NT5C3* | 5'-nucleotidase, cytosolic III |
| *PACRG* | PARK2 co-regulated |
| *PDIA5* | protein disulfide isomerase family A, member 5 |
| *PHYH* | phytanoyl-CoA 2-hydroxylase |
| *PKIA* | protein kinase (cAMP-dependent, catalytic) inhibitor alpha |
| *PLAC8* | placenta-specific 8 |
| *PPP1R13B* | protein phosphatase 1, regulatory subunit 13B |
| *POLD3* | polymerase (DNA-directed), delta 3, accessory subunit |
| *POLR1D* | polymerase (RNA) I polypeptide D, 16kDa |
| *PRPF6* | PRP6 pre-mRNA processing factor 6 homolog (S. cerevisiae) |
| *PSD3* | pleckstrin and Sec7 domain containing 3 |
| *PTTG1IP* | pituitary tumor-transforming 1 interacting protein |
| *PTX3* | pentraxin 3, long |
| *PURG* | purine-rich element binding protein G |
| *RBBP5* | retinoblastoma binding protein 5 |
| *RECQL5* | RecQ protein-like 5 |
| *RETNLB* | resistin like beta |
| *RNASE4* | ribonuclease, RNase A family, 4 |
| *RPL36P14* | ribosomal protein L36 pseudogene 14 |
| *RPUSD4* | RNA pseudouridylate synthase domain containing 4 |
| *SACS* | spastic ataxia of Charlevoix-Saguenay (sacsin) |
| *SAMD7* | sterile alpha motif domain containing 7 |
| *SBF2* | SET binding factor 2 |
| *SEC63* | SEC63 homolog (S. cerevisiae) |
| *SLAMF1* | signaling lymphocytic activation molecule family member 1 |
| *SLC7A2* | solute carrier family 7 (cationic amino acid transporter, y+ system), member 2 |
| *SNX12* | sorting nexin 12 |
| *SPESP1* | sperm equatorial segment protein 1 |
| *STAM2* | signal transducing adaptor molecule (SH3 domain and ITAM motif) 2 |
| *STT3B* | STT3, subunit of the oligosaccharyltransferase complex, homolog B (S. cerevisiae) |
| *STXBP4* | syntaxin binding protein 4 |
| *STX2* | syntaxin 2 |
| *SUPT3H* | suppressor of Ty 3 homolog (S. cerevisiae) |
| *TBC1D24* | TBC1 domain family, member 24 |
| *THAP1* | THAP domain containing, apoptosis associated protein 1 |
| *TH1L* | TH1-like (Drosophila) |
| *TMEM200A* | transmembrane protein 200A |
| *TOM1L1* | target of myb1 (chicken)-like 1 |
| *TOP2A* | topoisomerase (DNA) II alpha 170kDa |
| *TTC28* | tetratricopeptide repeat domain 28 |
| *UBQLN2* | ubiquilin 2 |
| *UMPS* | uridine monophosphate synthetase |
| *USH2A* | Usher syndrome 2A (autosomal recessive, mild) |
| *USP1* | ubiquitin specific peptidase 1 |
| *ZNF300* | zinc finger protein 300 |
| *ZNF619* | zinc finger protein 619 |
| *ZNF763* | zinc finger protein 763 |
