# Supplementary material for: A Synthetic Interaction Screen Identifies Factors Selectively Required for Proliferation and TERT Transcription in p53-Deficient Human Cancer Cells
Source: PLoS Genet. 2012 Dec 20;8(12):e1003151. doi: 10.1371/journal.pgen.1003151 (PMC3527276; doi:10.1371/journal.pgen.1003151)
Supplement: Table S2 — Summary of the cell culture results in Figure 1 and Figure 2. (DOC) [file pgen.1003151.s018.doc]

**Table S2** Summary of the cell culture results in Figures 1 and 2.

| Gene | Name | Function | Fold decreased proliferation in p53- relative to p53+ cells | | | Decreased proliferation in  NCI-H1299 and NCI-H522 relative to A549 and NCI-H460 |
| --- | --- | --- | --- | --- | --- | --- |
|  |  |  | HCT116 | RKO | A549 |  |
| *ATR* | ataxia telangiectasia and Rad3 related | serine/threonine protein kinase | 5.05 | 1.73 | 1.98 | + |
| *DCLRE1C* | DNA cross-link repair 1C | exonuclease/endonuclease involved in V(D)J recombination and DNA repair | 2.49 | 1.10 | NT | NT |
| *ETV1* | ets variant 1 | transcription factor | 3.57 | 2.25 | 2.44 | + |
| *GFPT2* | glutamine-fructose-6-phosphate transaminase 2 | glucose metabolism | 3.15 | 2.92 | 2.18 | – |
| *GOLGA2P5* | golgin A2 pseudogene 5 | unknown | 2.73 | 1.09 | NT | NT |
| *NT5C3* | 5'-nucleotidase, cytosolic III | dephosphorylates pyrimidine 5' monophosphates | 2.87 | 1.67 | 1.52 | – |
| *PPP1R13B* | protein phosphatase 1, regulatory subunit 13B | required for the induction of apoptosis by p53-family proteins | 2.79 | 1.01 | NT | NT |
| *RPUSD4* | RNA pseudouridylate synthase domain containing 4 | unknown | 3.15 | 0.87 | NT | NT |
| *SNX12* | sorting nexin 12 | may be involved in intracellular trafficking | 3.46 | 1.11 | NT | NT |
| *UBQLN2* | ubiquilin 2 | ubiquitin-like protein | 3.09 | 1.18 | NT | NT |
| *UMPS* | uridine monophosphate synthetase | pyrimidine biosynthesis | 4.11 | 2.71 | 2.87 | – |

+, decreased proliferation relative to p53+ cells; –, no significant change in proliferation relative to p53+ cells; NT, not tested.
