# Supplementary material for: A Synthetic Interaction Screen Identifies Factors Selectively Required for Proliferation and TERT Transcription in p53-Deficient Human Cancer Cells
Source: PLoS Genet. 2012 Dec 20;8(12):e1003151. doi: 10.1371/journal.pgen.1003151 (PMC3527276; doi:10.1371/journal.pgen.1003151)
Supplement: Table S3 — Basis for the p53− status in each of the p53− cell lines used in this study. (DOC) [file pgen.1003151.s019.doc]

**Table S3** Basis for the p53- status in each of the p53- cell lines used in this study.

| Cell line | Specific DNA alteration |
| --- | --- |
| p53- HCT116 | Deletion of exon 2 |
| p53- RKO | Deletion of exon 2 |
| p53- A549 | Ectopic expression of a murine p53 dominant-negative mutant lacking the transactivation and DNA-binding domains (∆ amino acids 15-301) |
| NCI-H1299 | Homozygous partial deletion |
| NCI-H522 | Frame-shift mutation (P191fs*57) |
| DLD-1 | Point mutation (Ser 241 Phe) |
| HT29 | Point mutation (Arg 273 His) |
| HeLa | Inactivation of wild-type p53 by human papilloma virus 18 (HPV-18) |
