# Supplementary material for: A Synthetic Interaction Screen Identifies Factors Selectively Required for Proliferation and TERT Transcription in p53-Deficient Human Cancer Cells
Source: PLoS Genet. 2012 Dec 20;8(12):e1003151. doi: 10.1371/journal.pgen.1003151 (PMC3527276; doi:10.1371/journal.pgen.1003151)
Supplement: Table S4 — Oligo ID numbers and locations for shRNAs obtained from Open Biosystems, sequences of synthesized siRNAs, and primer sequences for qRT-PCR analysis. (DOC) [file pgen.1003151.s020.doc]

**Table S4** Oligo ID numbers and locations for shRNAs obtained from Open Biosystems, sequences of synthesized siRNAs, and primer sequences for qRT-PCR analysis.

**shRNAs**

| **Gene** | **Oligo/Clone ID** |
| --- | --- |
| *ATR* | V2LHS_94661 |
| *ATR-1* | TRCN0000039613 |
| *ATR-2* | TRCN0000039614 |
| *DCLRE1C* | V2LHS_80738 |
| *ETV1* | V2LHS_192233 |
| *ETV-1* | TRCN0000013925 |
| *ETV1-2* | TRCN0000013927 |
| *GFPT2* | V2LHS_50315 |
| *GOLGA2P5* | V2LHS_154773 |
| *NT5C3* | V2LHS_193312 |
| *PPP1R13B* | V2LHS_75080 |
| *RPUSD4* | V2LHS_177570 |
| *SNX12* | V2LHS_65155 |
| *TERT-1* | V3LHS_340162 |
| *TERT-2* | V3LHS_340160 |
| *UBQLN2* | V2LHS_58285 |
| *UMPS* | V2LHS_92651 |

**siRNAs**

| **Gene** | **Strand** | **Sequence (5’ to 3’)** |
| --- | --- | --- |
| *ATR* | Sense | UAAGACACUCAUUAAAGGC EXT UC |
|  | Antisense | GCCUUUAAUGAGUGUCUUA EXT UU |
| *DCLRE1C* | Sense | GUGGGAAGUAUUCUUUAAA EXT UU |
|  | Antisense | UUUAAAGAAUACUUCCCAC EXT UU |
| *ETV1* | Sense | UAACUGUACCAUAGAUACC EXT CC |
|  | Antisense | GGUAUCUAUGGUACAGUUA EXT UU |
| *GFPT2* | Sense | UAUUGAAAACUGAUUCUGG EXT CU |
|  | Antisense | CCAGAAUCAGUUUUCAAUA EXT UU |
| *GOLGA2P5* | Sense | GCCGGAAUAAGAGCAAUGA EXT UU |
|  | Antisense | UCAUUGCUCUUAUUCCGGC EXT UU |
| *LMNA* | Sense | CUGGACUUCCAGAAGAACA EXT TT |
|  | Antisense | UGUUCUUCUGGAAGUCCAG EXT TT |
| *NT5C3* | Sense | UAAAGCUUGCUGAACAAGC EXT UU |
|  | Antisense | GCUUGUUCAGCAAGCUUUA EXT UU |
| *PPP1R13B* | Sense | GCCUUAAAUAAGUCAGUUA EXT UU |
|  | Antisense | UAACUGACUUAUUUAAGGC EXT GC |
| *RPUSD4* | Sense | CAGCCCAUCACUGGAAUAA EXT UU |
|  | Antisense | UUAUUCCAGUGAUGGGCUG EXT UU |
| *SNX12* | Sense | CGAGCAGUUUAUUAACAAA EXT UU |
|  | Antisense | UUUGUUAAUAAACUGCUCG EXT UU |
| *UBQLN2* | Sense | CCCGCUGUUUACUGCAAAU EXT UU |
|  | Antisense | AUUUGCAGUAAACAGCGGG EXT UU |
| *UMPS* | Sense | UUGAACAGAUAACUGUAGC EXT CA |
|  | Antisense | GCUACAGUUAUCUGUUCAA EXT UU |

**qRT-PCR Primers**

| **Gene** | **Primer sequence (5’ to 3’)** |
| --- | --- |
| *ATR* | For: AAGCGCCACTGAATGAAACT |
|  | Rev: AACGGCAGTCCTGTCACTCT |
| *DCLRE1C* | For: GTGGTTTGGAGAAAGGAGCA |
|  | Rev: TTGGATATGCGTTCACAGGA |
| *ETV1* | For: TAGCCGTTCACTCCGCTATT |
|  | Rev: TATCTGGAAAGGCCATGGAG |
| *GFPT2* | For: GGCTGTTCTCCGAGGATATG |
|  | Rev: TGTTGGGACAGGTCTGGAAT |
| *GOLGA2P5* | For: TGGAGAAGCTGTGGGAGAGT |
|  | Rev: TCACCTTCACCTCTCCCTTG |
| *NT5C3* | For: AAGAATGGCAGATGGAGTGG |
|  | Rev: ACAGTTCAATTGCACCCACA |
| *PPP1R13B* | For: GCCTCAACCATAAGCGACAT |
|  | Rev: CATCACACCCAGCTTTTCCT |
| *RPUSD4* | For: TCTCCACCAGGACAAGAACC |
|  | Rev: AGCATCTTTGCCAGGATAGG |
| *SNX12* | For: AGGACCTGACCGACGCTTAC |
|  | Rev: CGCAGGACTCCTTTAGCTTG |
| *TERT* | For: CGGTGTGCACCAACATCTAC |
|  | Rev: GGGTTCTTCCAAACTTGCTG |
| *UBQLN2* | For: CCAGCAGTTCATTCAGCAAA |
|  | Rev: TGAGCTGTTCCAGTTGTTGC |
| *UMPS* | For: GGCCACTGGGGACTACACTA |
|  | Rev: CCTGCTTCCAACTGAACTCC |
